# Toward understanding the genetic basis of adaptation to high-elevation life in poikilothermic species: A comparative transcriptomic analysis of two ranid frogs, *Rana chensinensis* and *R. kukunoris*

**DOI:** 10.1186/1471-2164-13-588

**Published:** 2012-11-01

**Authors:** Weizhao Yang, Yin Qi, Ke Bi, Jinzhong Fu

**Affiliations:** 1 Chengdu Institute of Biology, Chinese Academy of Sciences, Chengdu, 610041, China; 2 Graduate University of Chinese Academy of Sciences, Beijing, 100049, China; 3 Museum of Vertebrate Zoology, University of California, Berkeley, CA, 94720-3160, USA; 4 Department of Integrative Biology, University of Guelph, Guelph, Ontario, N1G 2W1, Canada

**Keywords:** Adaptation, High elevation, Transcriptome, Amphibian, Positive selection, Ka/Ks ratio, Candidate gene, Oxygen binding, UV radiation

## Abstract

**Background:**

Understanding how organisms adapt to high-elevation environments at a genome scale provides novel insights into the process of adaptive evolution. Previous studies have mainly focused on endothermic organisms, while poikilothermic species may have evolved different mechanisms to cope with high-elevation environments. In this context, we sequenced transcriptomes of a pair of closely related anuran species, *Rana chensinensis* and *R. kukunoris,* which inhabit respective low- and high-elevation habitats. By comparing the two transcriptomes, we identified candidate genes that may be involved in high-elevation adaption in poikilothermic species.

**Results:**

Over 66 million sequence reads from each transcriptome were generated. A total of 41,858 and 39,293 transcripts for each species were obtained by *de novo* assembly. By comparing the orthologous transcripts, we identified 125 protein-coding genes that have likely experienced strong positive selection (Ka/Ks>1). In addition, 335 genes that may bear a signature of positive selection (1≥Ka/Ks>0.5) were also recognized. By considering their functions, fourteen candidate genes were determined to be likely involved in high-elevation adaptation, including two CYP genes, USP-1, and several others.

**Conclusions:**

We identified a set of candidate genes that may have promoted adaptation of *R. kukunoris* to its high-elevation environment. These include several genes that have previously been associated with oxygen binding, response to UV radiation, and repair of free radical injury. Detailed molecular, physiological, and phenotypic studies are required to better understand the roles of these genes in improving the performance of *R. kukunoris* in a high-elevation environment. We have evidence for both convergent and divergent evolution between endothermic and poikilothemic species, but additional research across a wide range of organisms will be necessary to comprehend the complexity of high-elevation adaptation.

## Background

Elucidating the process of adaptation and understanding its genetic basis is a major mission of modern evolutionary biology [1,2]. Although there are a few genome-level studies of model organisms [3,4], most previous studies have focused on a single or a few candidate genes, which limited our understanding of the molecular basis of adaptation in many non-model systems. Recent advances in DNA sequencing technology and bioinformatic tools enable us to access and analyse massive sequence data in a short time at an affordable cost, which allows large-scale comparisons at genome, exome, or transcriptome levels [5,6]. These advances present new opportunities for examining the genetic basis of many adaptive processes.

Understanding how organisms adapt to high-elevation environments can provide important insights into the process of adaptive evolution, particularly interactions and trade-offs between genes and pathways involved in simultaneous adaptive responses to multiple environmental challenges [7-10]. High-elevation environments impose severe physiological challenges to organisms, particularly those related to declined levels of oxygen, low temperature, and elevated levels of ultraviolet (UV) radiation [11,12]. Physiological variation and adaptation to high-elevation habitats are well documented in many vertebrates [13-15]; however, study of their genetic basis is still in its infancy. Much of the previous work has focused on a few candidate loci, such as hemoglobin related genes and mitochondrial genes [16-18]. Only recently, analyses of genome-wide variations among species and populations living in different altitudinal environments have begun to shed light into the genetic basis of many adaptive processes. For example, genes related to hypoxia response and energy metabolism were shown to be responsible for physiological adaptations in several high-elevation species [9,10,19-21]. All these genome-wide studies investigated endothermic species. Ectothermic species have large physiological differences and may have evolved different mechanisms for adaption to high-elevation environments [12]. For example, instead of maintaining high levels of metabolism for temperature stability, poikilothermic species may lower their metabolic rate to cope with low external temperatures [12]. Understanding the genetic basis of adaptation to high elevations in poikilothermic species will not only complement our knowledge about endothermic species, but may also reveal novel mechanisms that are applicable to species that represent a much larger portion of the tree of life.

A pair of anuran species, *Rana chensinensis* and *R. kukunoris*, provide an excellent model system to study how poikilothermic species adapt to high-altitude habitats. *Rana chensinensis* is a widespread species and primarily occurs at low elevations of 600–1300 m above sea level (a.s.l.). *Rana kukunoris* is a high-elevation species that is only found at 2800–4000 m a.s.l. [22]. Previous studies suggest that the two species descended from a *R. chensinensis*-like common ancestor that inhabited low elevations, and speciation of *R. kukunoris* coincided with the recent uplift of the Tibetan Plateau approximately 7.8 million years ago [23]. Both species are diploid with 24 pairs of chromosomes [24] and *R. chensiensis* has a diploid genome size of 10.05 picograms (approximately 9,800 mega base pairs) [25].

The objective of this study is to make a genome wide search for genes that may be involved in adaptation to high-elevation environments in these amphibians, and identify their associated functions. We sampled multiple tissues of *R. chensinensis* and *R. kukunoris* and sequenced their transcriptomes using an Illumina sequencing platform. By comparing their transcriptomes, we describe genes showing strong signals of positive selection. The functional and phenotypic outcomes of these candidate genes are further inferred by annotating to genomic resources of a variety of model vertebrate species. We report a list of candidate genes that are highly likely to be involved in high-elevation adaptation processes.

## Results and discussion

### Illumina sequencing, *de novo* assembly, and gene annotation

A total of 67,676,712 sequence reads of *R. chensinensis*, and 66,476,534 sequence reads of *R. kukunoris*, were generated by Illumina sequencing. We first filtered these reads, and removed 527,878 and 711,299 defective reads for each species, respectively.

**Figure 1:**
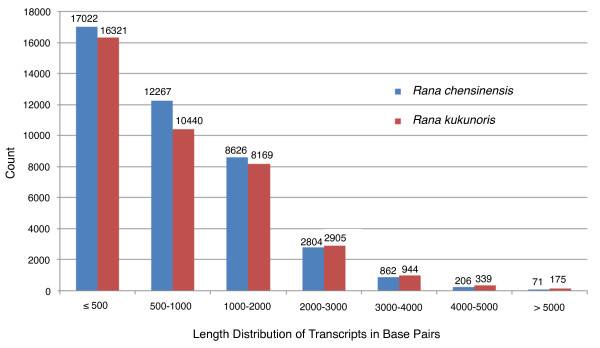
**Length distribution of transcripts in base pairs.** The numbers of transcripts are shown on top of each bar.

*De novo* assembly of cleaned reads was performed using various combinations of K-mer lengths and coverage cut-off values [26,27]. In total, 30 raw assemblies were obtained for each species and were further merged by integrating sequence overlaps and eliminating redundancies for each species. The total length of the final set of assemblies was 38.1 mega base pairs (Mb) for *R. chensinensis* and 37.6 Mb for *R. kukunoris*. For *R. chensinensis*, 41,858 transcripts were obtained with an N50 of 1,333 base pairs (bp) and a mean length of 909 bp, and for *R. kukunoris*, 39,293 transcripts were obtained with an N50 of 1,485 bp and a mean length of 956 bp. All information of data production is summarized in Table 1, and the length distribution of all transcripts is shown in Figure 1. 

**Table 1:** **Summary of transcriptome data for *Rana chensinensis *and *R. kukunoris***

	***R. chensinensis***		***R. kukunoris***	
	**raw assemblies**	**final assembly**	**raw assemblies**	**final assembly**
Total number of reads	67,676,712		66,476,534	
Read length (bp)	90		90	
Total length of reads (bp)	6,090,904,080		5,982,888,060	
Total length of assembly (Mb)	742.5	38.1	751.4	37.6
Total sequences of assembly	1,624,822	41,858	1,521,607	39,293
N50 length of assembly (bp)	515	1,333	586	1,485
Mean length of assembly(bp)	456	909	493	956
Median length of assembly(bp)	325	599	340	601
Total number of transcripts	41,858		39,293	
Transcript annotated	16,738		16,549	
Number of unique genes represented	11,179		11,140	
Number of putative orthologs	7069			

bp=base pair; Mb=mega base pairs.

We constructed a reference dataset using combined protein data from seven species that represented all major lineages of vertebrates for gene annotation. Transcripts that did not match the reference dataset were blasted against a non-redundant (NR) protein database of the GenBank. In total, 16,738 transcripts of *R. chensinensis* were annotated to 11,179 unique genes, accounting for 40.0 % of the total transcripts. Some transcripts were likely products of alternative splicing. The E-value distribution showed that 55.3% of the annotated transcripts had an E-value below 1E-50, and the similarity distribution showed that 59.2% of the annotated transcripts had a similarity greater than 60%. Similarly, 16,549 transcripts of *R. kukunoris* were annotated to 11,140 unique genes, accounting for 42.1% of the total transcripts. Furthermore, 56.6% of the annotated transcripts had an E-value below 1E-50, and 59.7% of the annotated transcripts had a similarity greater than 60% (Table 1 and Additional file 1).

In agreement with previous studies, we found that the quality of sequence reads and appropriate assembly methods are essential in obtaining a reliable *de novo* assembly, which is the foundation of all other analyses [26,28,29]. In particular, *de novo* assembly by exploring a wide range of K-mer and cut-off values is a powerful approach. Due to the differential gene expression, sequence coverage among transcripts tends to be uneven, which poses various challenges for *de novo* assembly [26,27]. The use of high K-mer lengths can generate more contiguous assemblies of highly expressed transcripts. On the other hand, the use of low K-mer lengths likely helps to obtain better assemblies for under expressed genes [26]. Adjusting coverage cut-off value can also help to increase the assembly quality when there are similar regions among homologous transcripts [27]. We used five different K-mer lengths and six different coverage cut-off values. In both species, the final assembly had larger N50 and mean lengths than any of the raw assemblies generated by using a single value of both parameters (Table 1). The final assembly also had a higher proportion of annotated transcripts (~40%) than all raw assemblies. Moreover, a final step that maps all reads back to the assembled transcripts can also help to improve the quality of assembly. This process allows for a revision of any potential assembly errors and ascertains the consensus sequences representing the majority base calls at heterozygous sites.

### Gene ontology (GO) classification

Gene Ontology category was widely used to classify gene functions [30]. We first removed 5,559 transcripts for *R. chensinensis* and 5,409 transcripts for *R. kukunoris* that were likely generated from alternative splicing. Among the remaining 11,179 transcripts of *R. chensinensis,* which likely represented the same number of unique genes, a total of 8,781 transcripts had the GO annotation under three main divisions. Likewise, among the 11,140 annotated transcripts of *R. kukunoris*, 8,806 transcripts had the GO annotation. The GO category distributions of the transcripts for both species were highly similar ( Additional file 2). Within the function of “Cellular Component”, 13 level-2 categories were identified, and the terms “cell”, “cell part”, and “organelle” were the dominant components (>50%). Within the function of “Molecular Functions”, 15 level-2 categories were identified, and the term “binding” was dominant (>50%). Within the third function of “Biological Process”, 23 level-2 categories were identified, and the terms “cellular process”, “metabolic process”, “biological regulation”, and “pigmentation” were the main functional categories (>50%).

### Identification of putative orthologs

Reciprocal best hit method was used to identify putative orthologs between the two species [31]. A total of 10,132 pairs of putative orthologs were identified by comparing all annotated transcripts. We then compared each pair of putative orthologs to the reference to determine their open reading frames (ORF) and eliminated the sequences with unexpected stop-codons and ambiguous alignments. After filtering the redundant orthologs that may result from alternative splicing, 7,069 pairs of unique orthologs were finally identified and used in the downstream analyses.

We also identified 8,393 pairs of possible orthologs from transcripts that could not be annotated. We were able to use EBOSS-GETORF [32] to predict the ORFs of 3,880 and 3,851 transcripts in *R. chensinensis* and *R. kukunoris*, respectively. Among these transcripts, 1,701 pairs were properly aligned, which were also used in the test for positive selection.

### Test for positive selection

The Ka/Ks ratio was used to test for positive selection [33]. Among the 7,069 pairs of annotated orthologs, 724 pairs were identical, 2,827 pairs had only synonymous or non-synonymous substitutions, and 3,518 pairs had both types of substitutions, for which the Ka/Ks ratio were calculated. The mean values of Ka, Ks, and Ka/Ks ratio of all orthologous pairs were 0.0054, 0.0372, and 0.1452, respectively.

**Figure 2:**
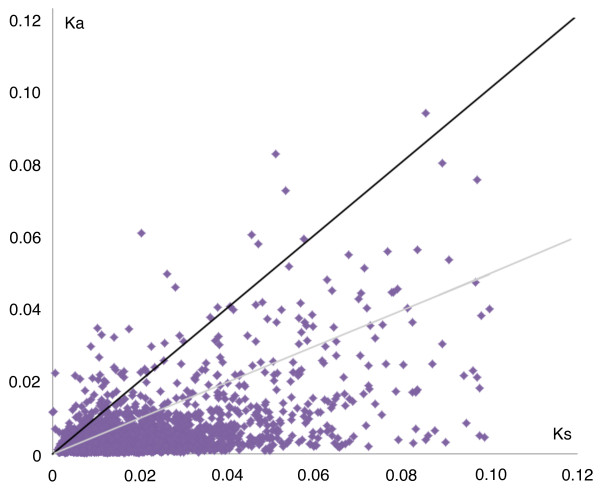
**Distribution of Ka and Ks values.** Orthologous pairs with Ka/Ks ratio >1 are above the black line; pairs with Ka/Ks ratio between 0.5-1 are between the black and grey lines.

A total of 125 (1.8%) pairs of orthologs had a Ka/Ks ratio greater than 1, indicating these genes may have experienced strong positive selection (Figure 2). In addition, a Ka/Ks ratio of 0.5 was also suggested as a useful cut-off value to identify genes under positive selection [34], and in our study, 335 (4.7%) pairs of orthologs had a Ka/Ks ratio between 0.5 and 1 (Figure 2). Therefore, all these 460 orthologs (Ka/Ks>0.5) were considered as candidate genes that have likely experienced positive selection. 

Among the 1,701 pairs of predicted coding sequences from un-annotated transcripts, 25 (1.5%) pairs had a Ka/Ks ratio greater than 1 and 205 (12.0%) pairs had a Ka/Ks ratio between 0.5 and 1. They were supplementary to the annotated candidate gene list for future studies.

We clustered all the unique orthologous transcripts to GO categories. Among the 7,069 annotated unique orthologs, 6,811 were assigned to 8,335 GO terms, among which 1,143 terms had at least 10 orthologs. The mean Ka/Ks ratio for each of the 1,143 terms was calculated ( Additional file 3), and 330 (28.9%) terms had a mean Ka/Ks ratio higher than the mean ratio of all orthologs (0.1452). The terms with the highest Ka/Ks ratio included “adult heart development”, “sarcomere organization” and “condensed nuclear chromosome”. Likewise, the terms with the smallest Ka/Ks ratio included “protein K63-linked ubiquitination”, “protein K48-linked ubiquitination” and “ubiquitin-dependent protein catabolic process”. The terms with low Ka/Ks ratio probably represent the most conservative functions, and the terms with high Ka/Ks ratio likely represent the rapidly evolving functions between the two species [10,35].

### Functional analysis for candidate genes

**Figure 3:**
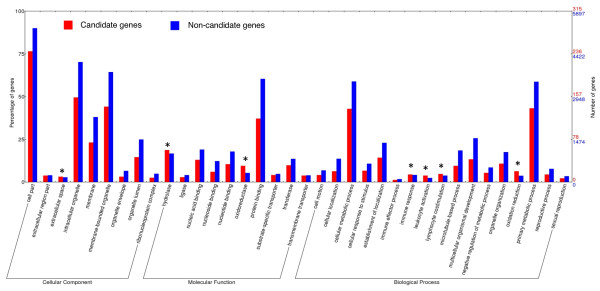
**Comparison of Gene Ontology (GO) category (level 3) distribution between candidate genes and non-candidate gene. ** The red and blue bars represent candidate genes (Ka/Ks ratio >0.5) and non-candidate genes (Ka/Ks ratio <0.5) in each GO functional category, respectively. Candidate genes have higher percentage then non-candidate gene in seven categories, which are marked with asterisks. The sum of individual percentages exceeds 100% because most genes are classified in multiple categories.

Among the 460 candidate genes (Ka/Ks >0.5), 315 were annotated to GO categories (68.5%). Using the program WEGO [36], we compared the GO category distribution (level 3) of the candidate genes with that of the non-candidate genes (Ka/Ks < 0.5) (Figure 3). Seven GO terms had a higher percentage among the candidate genes than among the non-candidate genes. Several of these terms were associated with immunity and host defense, such as “immune response” (4.4% among candidate genes vs. 4.2% among non-candidate genes), “leukocyte activation” (3.8% vs. 2.4%), and “lymphocyte costimulation” (4.8% vs. 3.8%). Immune related genes were commonly found under positive selection during speciation processes and in species that do not live at high elevations [35,37]. Therefore, although under positive selection, these genes may not be related to adaptation to a high-elevation environment. 

To identify genes that may closely associate to the adaptation to high elevations, we performed a two-step approach to screen all the candidate genes according to their functions. First, we compared our candidate genes to a list proposed by Simonson *et al*. [20], which encompasses the functions and their associated GO categories for adaptation to high-elevation hypoxia. Two genes associated with “response to hypoxia” and two genes associated with “oxygen binding” were identified from our candidate gene list (Table 2). Second, based on our current understanding on phenotypic adaptation to high elevations of poikilothermic species, we selected three other candidate functions, which are associated with response to ultraviolet rays [11], reactive oxygen species metabolism, and metabolic regulation [12]. Ten additional genes were identified with these candidate functions, which were in the GO categories “response to UV”, “glutathione metabolic process”, and “ATP catabolic process” (Table 2). 

**Table 2:** List of fourteen candidate genes with related Gene Ontology (GO) categories

**Sequence ID**	**Protein homolog**	**Ka/Ks ratio**	**GO category**	**GO accession**
Kuku_seq_14688	Glutathione s-transferase omega 2, GSTO2	1.7284	glutathione metabolic process	GO:0006749
Kuku_seq_38792	Sterol carrier protein 2, SCP2	1.4657	peroxisome organization	GO:0007031
Kuku_seq_19028	Ubiquitin-specific protease 1, USP-1	1.2268	response to UV	GO:0009411
Kuku_seq_38041	Ribosomal L1 domain containing 1,RSL1D1	1.1671	primary metabolic process	GO:0044238
Kuku_seq_19815	Peroxiredoxin-4, PRDX4	1.1484	response to oxidative stress	GO:0006979
Kuku_seq_9362	Mitochondrial translation optimization 1, MTO-1	1.1363	mitochondrial tRNA wobble uridine modification	GO:0070899
Kuku_seq_22135	NADPH oxidase organizer 1, NOXO1	1.0432	NADPH oxidase complex	GO:0043020
Kuku_seq_29724	Aminoacylase-1, ACY1	1.0342	protein catabolic process	GO:0030163
Kuku_seq_10530	DNA repair protein xrcc, XRCC	0.6313	response to hypoxia	GO:0001666
Kuku_seq_19044	4-aminobutyrate aminotransferase, ABAT	0.5899	response to hypoxia	GO:0001666
Kuku_seq_19251	Cytochrome p450 4b1-like, CYP4B1	0.5867	oxygen binding	GO:0019825
Kuku_seq_4377	Glutathione peroxidase 4, GPX4	0.5799	glutathione metabolic process	GO:0006749
Kuku_seq_29173	V-type proton ATPase subunit G 1, V-ATPase G1	0.5173	ATP catabolic process	GO:0006200
Kuku_seq_8042	Cytochrome p450 2f1-like, CYP2F1	0.5062	oxygen binding	GO:0019825

These fourteen genes have Ka/Ks ratio greater than 0.5 and are most likely involved in the adaptation to high-elevation environments.

The two candidate genes associated with “oxygen binding”, CYP2F1 and CYP4B1, belong to the cytochrome P450 superfamily (CYP). The function of CYP is to catalyze the oxidation of organic substances [38]. The mean Ka/Ks ratio of the term “oxygen binding” is 0.2435, much higher than the mean Ka/Ks ratio of all orthologs (0.1452), suggesting a strong overall positive selection on genes in this category. Previous studies of Tibetan people also found that several other CYP genes showed signatures of positive selection related to “oxygen binding” [20]. Our finding suggests a functional (phenotypic) convergent evolution between humans and *Rana kukunoris*, although the genes behind the functional adaptation are different (but belong to the same gene family).

The gene Ubiquitin-specific protease 1 (USP-1) is also under strong positive selection (Ka/Ks=1.2268), and we found a non-synonymous substitution in its His domain of N-terminal, which is likely crucial for its function [39]. USP-1 is a conserved gene that encode an enzyme playing an important role in DNA repair against UV damage [39,40]. Thus, USP-1 may be especially important for amphibians to adapt to the high UV radiation environment, since their skin is completely exposed without any protection from fur or scales.

Four genes, GPX4, GSTO2, PRDX4, SCP2, which are associated with response to reactive oxygen species (ROS), have also experienced positive selection (Table 2). The first two genes encode crucial enzymes in glutathione metabolic pathway, which prevent damage to important cellular components by ROS-like peroxides and free radicals [41]. The functions of the latter two genes are related to the functions of peroxisome that are also associated with response to oxidative stress [42,43]. Bickler and Buck [12] proposed that the ability of poikilothermic animals to survive under low temperature and oxygen conditions depends on the ability of avoiding free-radical injury. The four genes identified here are potentially related to the adaptive process in this aspect.

We also identified five metabolism related genes that may be associated with adaptation to high elevations. The MTO-1 and V-ATPase G1 genes are related to ATP synthesis and energy metabolism in the mitochondrion [44,45]; both functions are important in the process of high-elevation adaptation [12]. The ACY1, NOXO1 and RSL1D1genes are related to primary, NADPH and protein metabolism, respectively [46-48]. Functional changes associated with metabolic processes are likely essential for the adaptation to high-elevation environments.

A surprising absence from our candidate gene list is the hemoglobin related genes. Responses to hypoxia in endothermic animal species involve mostly hemoglobin genes and HIF-1 pathways, and we did not observe any of those genes bearing signature of positive selection. The mean Ka/Ks ratio (0.1154) of the entire term “response to hypoxia” was lower than average level (0.1452), although two genes (ABAT and XRCC) had Ka/Ks ratio greater than 0.5 but less than 1 (Table 2). Some other genes involved in adaptation to high elevations in endothermic species also had a low Ka/Ks ratio in our comparison, for example, EPAS1 [20,21] (Ka/Ks=0.1057), ARG2 [10] (Ka/Ks=0.1095), and EP300 [19] (Ka/Ks=0.1725). The gene EGLN1 [19] is an exception in this sense; it has a relatively high Ka/Ks ratio of 0.4563, which may deserve further examination. This observation is consistent with our prediction that poikilothermic species may develop different adaptive mechanisms from endotherms.

### Future directions

Our study demonstrated that comparing sequences of orthologous genes between species by *de novo* transcriptome sequencing offers a fast and cost-effective approach to detect positive selection and to understand adaptive evolution at the genomic level. Further tests are needed to validate these candidate genes that we have identified. Population-level analysis can be applied to these candidate genes to examine if signals of positive selection on these genes are also evident at the population level. Detailed functional research is also needed to illustrate how the positively selected genes promote the fitness of poikilothermic species under the high-elevation environment. Finally, to understand the general genetic mechanisms of adaptation to high elevations of poikilothermic animals, it is necessary to examine other poikilothermic species from various groups. For example, hemoglobin genes, which were thought to play an important role in adaptation to high elevations in endothermic species, were absent from our candidate gene list and the importance of their role remained largely unknown in other poikilothermic species [17]. Studies on a wide range of species are necessary to understand parallel and convergent adaptive evolution to high elevations between poikilotherms and endotherms.

Better and more elaborative tests for positive selection should be explored in order to improve their sensitivity. Ka/Ks ratio can only serve as a preliminary approximate estimator. Functional analysis with pre-defined “potential functions for adaptation”, or candidate genes, largely limits the scope of discovery. Only with tests that are solely based on genetic data at a genome-wide scope, it is possible to establish a new ‘genotype to phenotype’ approach to the study of adaptation, which will complement the traditional ‘phenotype to genotype’ approach. With this new approach, we can take the great advantage of rapidly accumulating genomic data to reveal novel phenotypes of adaptation.

## Conclusions

By comparing the transcriptomes of two ranid species, one from high and one from low altitudes, we identified 125 protein-coding genes that have experienced strong positive selection (Ka/Ks>1), and 335 genes that may also bear signatures of positive selection (1≥Ka/Ks>0.5). Among them, 14 genes are most likely involved in the adaptive process to high-elevation environments, particularly genes associated with oxygen binding (CYP4B1and CYP2F1), response to UV (USP-1), and repair of free radical injuries (GSTO2, GPX4, PRDX4, and SCP2). Furthermore, we found evidence for convergent evolution between poikilothermic and endothermic species; genes from the CYP superfamily are under positive selection in both humans and amphibians. Yet, we also observed differences between them; hemoglobin related genes are commonly involved in high-elevation adaptation in endotherms, but are absent from our candidate gene list. The genes that display signatures of positive selection will serve as a baseline for further investigations that aim to understand high-elevation adaptation at both molecular and phenotypic levels.

## Methods

### Sample collection

To include as many expressed genes as possible, we sampled five different types of tissues from six individuals of each species, including liver, heart, skeletal muscle, testicle/ootheca from two males and two females, and the bodies of two tadpoles. Samples of *R. chensinensis* were collected in April, 2011, from the Yaodu Township, Qingchuan County, located on northern Sichuan Basin, China (105.45°E, 32.79°N) with an altitude of 604 m above sea level (a.s.l.). Samples of *R. kukunoris* were collected in April, 2011, from the Zoige County, situated on the eastern margin of the Tibetan Plateau, China (102.90°E, 33.58°N) with an altitude of 3,358 m a.s.l. Sample locations are depicted on a map in Additional file 4. Tissue samples for RNA extraction were stored in RNALater (QIAGEN) immediately following euthanasia and dissection.

### cDNA library construction and Illumina sequencing

RNA extraction, cDNA library construction, and sequencing were performed by BGI (Shenzhen, China). The entire process followed a standardized procedure and was monitored by BGI’s Quality Control System. For each species, total RNA was extracted using SV Total RNA Isolation System (Promega) from each tissue sample independently and then mixed with approximately the same quantity. RNAs with poly (A) tail were purified from total RNA by oligonucleotide (dT) magnetic beads and interrupted into short sequences. Subsequently, cDNAs were synthesized and purified with PCR extraction kit (QiaQuick). The cDNA library had an insert size of 200–700 base pair (bp), and paired-end of the library was sequenced with a read length of 90 bp using the Illumina HiSeq™ 2000 sequencing platform. Output of image data from the sequencer was transformed by base calling into raw sequence data, which formed the raw reads and then stored in the FastQ format.

### Data filtration and *de novo* assembly

We first cleaned the raw sequence reads by removing the exact duplicates from both sequencing directions. We further trimmed reads by removing adapter sequences, reads with unknown bases call (N) more than 5%, low complexity, and low quality (<Q20) using Blat [49]. We also removed reads likely derived from contaminants by comparing with the human and *Escherichia coli* genomes using Bowtie [50].

*De novo* assembly of cleaned reads was performed using a combination of various multiple K-mer lengths and coverage cut-off values [26,27]. Using VELVET [51] and OASES [52], five different K-mer lengths (21, 31, 41, 51, and 61) and six coverage cut-off values (2, 3, 6, 10, 15, and 20) were used to produce a total of 30 raw assemblies for each transcriptome. We then used CD-HIT-EST [53] and CAP3 [54] to produce a final assembly by integrating sequence overlaps and eliminating redundancies. All reads were mapped back to the assembled contigs using SOAP-ALIGNER [55] and SNPs were identified using SOAP-SNP [56]. For all variable sites, the base call with the most mapped reads was selected as the consensus using an in-house Python script.

### Gene annotation

Due to the lack of genomic resources from any *Rana* species, we combined protein data from seven species covering all major vertebrate groups to create a reference dataset for gene annotation, which includes *Anolis carolinensis*, *Danio rerio*, *Gallus gallus*, *Homo sapiens*, *Mus musculus*, *Oryzias latipes*, and *Xenopus tropicalis*[57]. All protein data were downloaded from the Ensembl database [58]. Transcripts were annotated to the reference dataset based on the BLAST similarity using blastx with an E-value cut-off of 1E-5. Transcripts that did not match to any gene in the reference dataset were searched against the non-redundant (NR) protein database of GenBank.

### Gene ontology classification

Gene Ontology [30] categories were used to classify the functions of the transcripts. In order to exclude the interference from alternative splicing of transcripts, we first clustered the transcripts that matched to the same reference gene; then we removed the redundant transcripts and only reserved the longest transcript for each cluster to represent the unique gene. GO classification was performed following the Blast2GO pipelines [59], based on a standard cut-off E-value of 1E-3 and a maximum of 20 hits of the BLAST process, and the default parameters of mapping and annotation procedure.

### Identification of putative orthologs

A reciprocal best hit method was used to identify putative orthologs between the two species with tblastx of BLAST-2.2.26+ and a bit-score threshold of 300 [31]. Subsequently, sequences in each orthologous pair were compared to the combined protein reference to determine their open reading frames (ORF) by blastx. The coding region of both sequences was then extracted and aligned by MUSCLE [60]. Aligned sequences with unexpected stop-codons, ambiguous alignments, and/or shorter than 200 bp were discarded from further analysis. A manual check was also conducted to correct potential errors. All transcripts, including products of potential alternative splicing, were blasted to maximize the chance of finding matches between the two species. If any orthologs that matched to the same reference gene had overlapping annotated region, we removed the shorter sequences to ensure that all orthologous pairs represented unique genes. For contigs that could not be annotated, we also applied the same method to identify orthologous pairs, then used EMBOSS-GETORF to predict the ORF for each sequence [32]. We adopted a strict criterion that only reserved the predicted coding sequence longer than 300 bp of each sequence. The resulting sequence pairs were analysed following the same approach described above.

### Test for positive selections

The ratio of the number of nonsynonymous substitutions per nonsynonymous site (Ka) to the number of synonymous substitutions per synonymous site (Ks) was used to test for positive selection. A Ka/Ks ratio less than one indicates purifying selection, while a Ka/Ks ratio greater than one provides evidence for positive selection [33]. For its simplicity and applicability, the ratio has been widely used in genome wide comparative studies, despite its limitations. Yang and Bielawski [33] provided a thorough evaluation of the application of Ka/Ks for testing selection. We employed the software KaKs_CALCULATOR [61] to estimate the Ka, Ks and Ka/Ks ratio of each orthologous pair, and the YN model [62] was used. The sequences with Ks >0.1 were excluded to avoid potential paralogs [63]. Based on the GO classification, the unique orthologs were clustered into different functional terms and the mean Ka/Ks ratio for each term was also calculated.

### Functional analysis for candidate genes

The orthologs that had Ka/Ks ratio above 0.5 were considered as candidate genes [34]. To detect which functions were more likely under positive selection, we compared the GO distribution (level 3) of the candidate genes with distribution of the non-candidate genes (Ka/Ks < 0.5) by the program WEGO [36]. Furthermore, we performed a two-step approach to identify candidate genes that might have been involved in facilitating the adaptation process to high-elevation environments. First, we examined the list of a priori functional candidate genes and the associated GO categories made by Simonson *et al*. [20], which represents the functions for adaptation to high elevation hypoxia and was used to screen candidate genes identified from a Tibetan Chinese population [20]. Second, based on previous studies on poikilotherms, we also studied other GO categories and selected the terms potentially associated with response to ultraviolet rays (UV), reactive oxygen species metabolism and metabolic regulation, which may represent other functions that have likely facilitated the adaptation to high elevations [11,12,64].

### Availability of supporting data

The data supporting the results of this article are available in the NCBI Sequence Read Archive (SRA) repository [Accession Number: SRA060325].

## Abbreviations

ACY1: Aminoacylase-1; ARG2: Arginase, type II; bp: Base pair; CYP2F1: Cytochrome p450 2f1-like; CYP4B1: Cytochrome p450 4b1-like; CYP: Cytochrome P450 superfamily; EGLN1: Egl nine homolog 1; EP300: E1A binding protein p300; EPAS1: Endothelial PAS domain-containing protein 1; GO: Gene Ontology; GPX4: Glutathione peroxidase 4; GSTO2: Glutathione s-transferase omega 2; Mb: Mega base pairs; MTO-1: Mitochondrial translation optimization 1; NOXO1: NADPH oxidase organizer 1; ORF: Open reading frame; PRDX4: Peroxiredoxin-4; ROS: Reactive oxygen species; RSL1D1: Ribosomal L1 domain containing 1; SCP2: Sterol carrier protein 2; USP-1: Ubiquitin-specific protease 1; UV: Ultraviolet; V-ATPase G1: V-type proton ATPase subunit G 1; XRCC: DNA repair protein xrcc.

## Competing interests

The authors declare that they have no competing interests.

## Authors’ contributions

WY carried out most of the data analyses and drafted the manuscript. YQ collected the specimens. KB participated in data analysis and writing. JF conceived the project and finalized the manuscript. All authors contributed to the project design and manuscript preparation. All authors have read and approved the final manuscript.

## Authors’ information

WY is a Ph.D. candidate and this project is part of his dissertation work. He is mostly interested in bioinformatics and related genomic work. YQ is a herpetologist and mostly interested in high elevation herpeto-fauna. KB is a post-doctoral fellow who is interested in detecting genome-wide signatures of natural selection using museum historical specimens. JF is an evolutionary biologist particularly interested in evolutionary phenomena around the Tibet Plateau.

## Supplementary Material

Additional file 1 **Characteristics of gene annotation of assembled transcripts against the reference dataset.** A. E-value distribution of blastx hits for each transcript with a cut-off E-value of 1E-5. B. Similarity distribution of blastx hits for each transcripts. Click here for file

Additional file 2 **Distribution of Gene Ontology (GO) categories (level 2) of transcripts for *Rana chensinensis* and *R. kukunoris.*** The GO functional annotations are summarized in three main categories: cellular component, molecular function and biological process. The red bars and blue bars represent *R. chensinensis* and *R. kukunoris*, respectively. (PDF 240 kb) Click here for file

Additional file 3 **Average Ka/Ks ratio for each GO term.** Only terms that have more than 10 orthologs are presented. Click here for file

Additional file 4 **Sampling sites and altitude distribution.** Samples of *Rana chensinensis* were collected from Yaodu Township (105.45°E, 32.79°N) with an altitude of 604 m above sea level. Samples of *R. kukunoris* were collected from Zoige County (102.90°E, 33.58°N) with an altitude of 3,358 m above sea level. Click here for file
