# Supplementary material for: Toward understanding the genetic basis of adaptation to high-elevation life in poikilothermic species: A comparative transcriptomic analysis of two ranid frogs, Rana chensinensis and R. kukunoris
Source: BMC Genomics. 2012 Nov 1;13:588. doi: 10.1186/1471-2164-13-588 (PMC3542248; doi:10.1186/1471-2164-13-588)

Additional file 1. Characteristics of gene annotation of assembled transcripts against the reference dataset. A. E-value distribution of blastx hits for each transcript with a cut-off E-value of 1E-5. **B.** Similarity distribution of blastx hits for each transcripts.

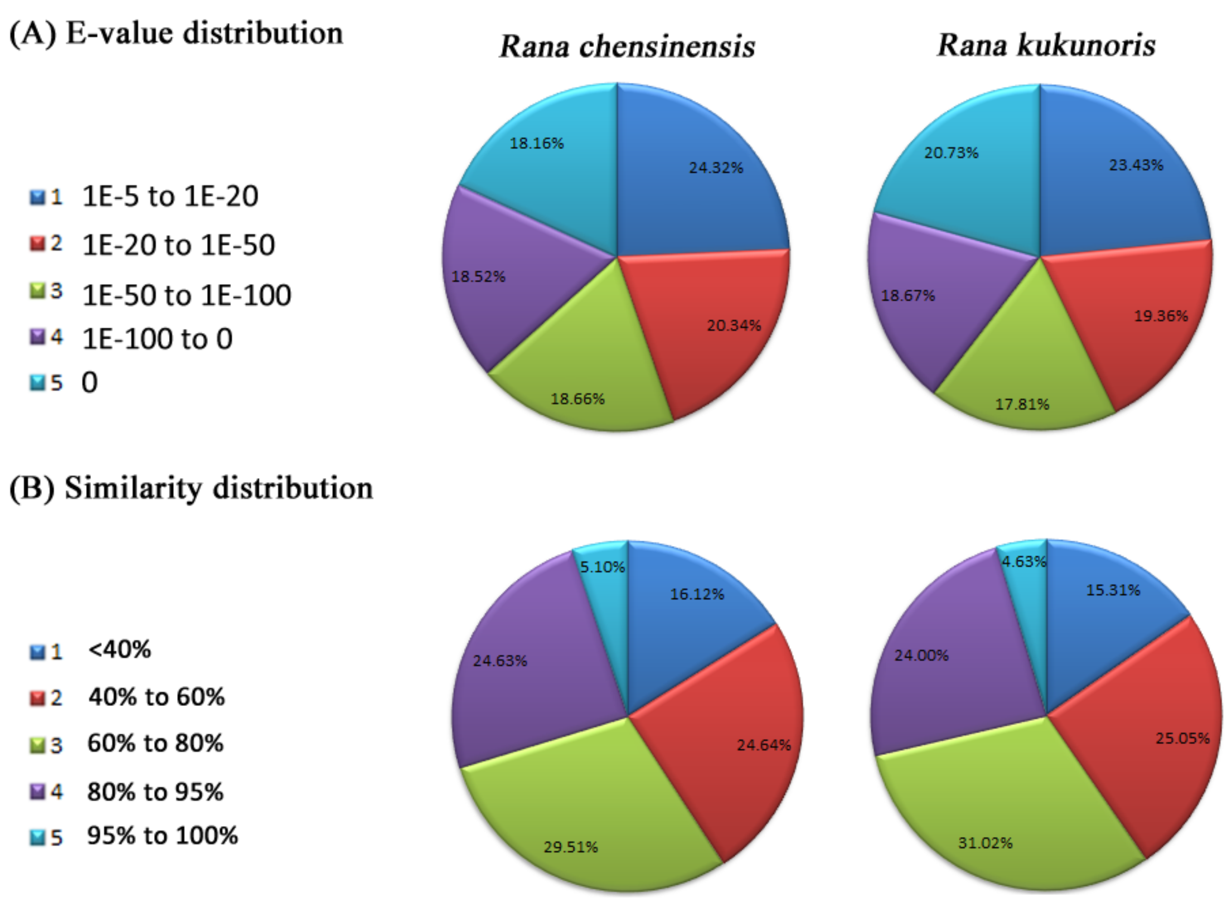

Supplement: Additional file 1 — Characteristics of gene annotation of assembled transcripts against the reference dataset. A. E-value distribution of blastx hits for each transcript with a cut-off E-value of 1E-5. B. Similarity distribution of blastx hits for each transcripts. [file 1471-2164-13-588-S1.pdf]
