# Supplementary material for: Toward understanding the genetic basis of adaptation to high-elevation life in poikilothermic species: A comparative transcriptomic analysis of two ranid frogs, Rana chensinensis and R. kukunoris
Source: BMC Genomics. 2012 Nov 1;13:588. doi: 10.1186/1471-2164-13-588 (PMC3542248; doi:10.1186/1471-2164-13-588)

Additional file 2. Distribution of Gene Ontology (GO) categories (level 2) of transcripts for *Rana chensinensis* and *R. kukunoris*. The GO functional annotations are summarized in three main categories: cellular component, molecular function and biological process. The red bars and blue bars represent *R. chensinensis* and *R. kukunoris*, respectively.

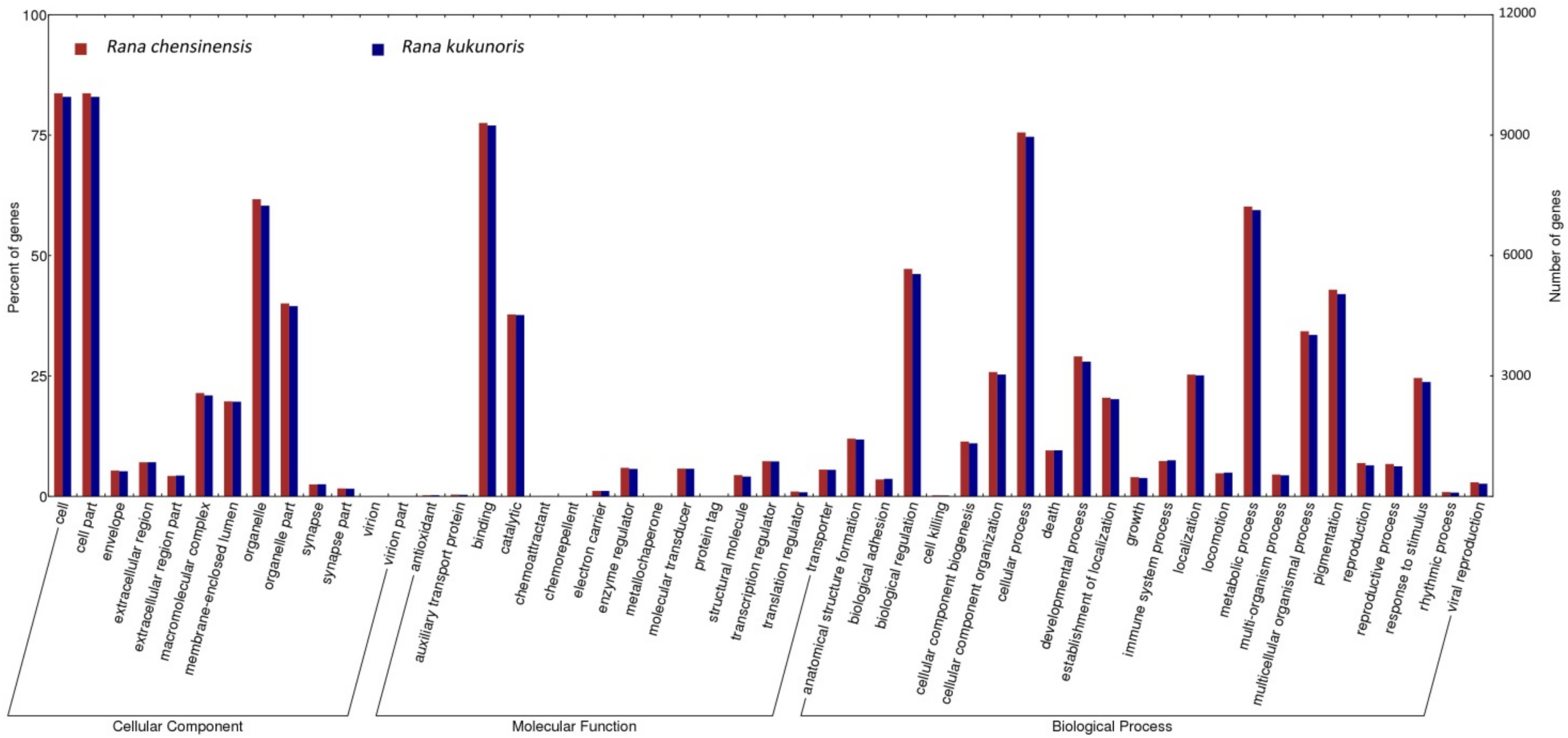

Supplement: Additional file 2 — Distribution of Gene Ontology (GO) categories (level 2) of transcripts for Rana chensinensis and R. kukunoris. The GO functional annotations are summarized in three main categories: cellular component, molecular function and biological process. The red bars and blue bars represent R. chensinensis and R. kukunoris, respectively. (PDF 240 kb) [file 1471-2164-13-588-S2.pdf]
