# Supplementary material for: Toward understanding the genetic basis of adaptation to high-elevation life in poikilothermic species: A comparative transcriptomic analysis of two ranid frogs, Rana chensinensis and R. kukunoris
Source: BMC Genomics. 2012 Nov 1;13:588. doi: 10.1186/1471-2164-13-588 (PMC3542248; doi:10.1186/1471-2164-13-588)
Supplement: Additional file 3 — Average Ka/Ks ratio for each GO term. Only terms that have more than 10 orthologs are presented. [file 1471-2164-13-588-S3.pdf]

Additional file 3. **Average Ka/Ks ratio for each GO term.** Only terms that have more than 10 orthologs are presented.

Gene Ontology Terms	Number of Unique Orthologs	Average Ka/Ks
GO:0007512 adult heart development	11	1.090567
GO:0045214 sarcomere organization	14	0.896056
GO:0000794 condensed nuclear chromosome	11	0.85024
GO:0019083 viral transcription	36	0.659227
GO:0043621 protein self-association	19	0.65417
GO:0006415 translational termination	39	0.648408
GO:0031018 endocrine pancreas development	44	0.64317
GO:0006414 translational elongation	48	0.614428
GO:0006919 activation of cysteine-type endopeptidase activity involved in apoptotic process	24	0.582188
GO:0016529 sarcoplasmic reticulum	10	0.566942
GO:0007076 mitotic chromosome condensation	10	0.565091
GO:0003735 structural constituent of ribosome	68	0.564691
GO:0010843 promoter binding	18	0.562071
GO:0005791 rough endoplasmic reticulum	12	0.552158
GO:0008307 structural constituent of muscle	36	0.539259
GO:0031526 brush border membrane	13	0.522147
GO:0048545 response to steroid hormone stimulus	15	0.506579
GO:0048731 system development	14	0.497494
GO:0051592 response to calcium ion	30	0.491538
GO:0005929 cilium	12	0.484441
GO:0044459 plasma membrane part	25	0.481713
GO:0080090 regulation of primary metabolic process	11	0.473547
GO:0051092 positive regulation of NF-kappaB transcription factor activity	26	0.441501
GO:0005793 endoplasmic reticulum-Golgi intermediate compartment	24	0.441172
GO:0048513 organ development	34	0.430245
GO:0042383 sarcolemma	39	0.417355
GO:0008202 steroid metabolic process	19	0.416856
GO:0022627 cytosolic small ribosomal subunit	20	0.416774
GO:0033344 cholesterol efflux	10	0.410206
GO:0005159 insulin-like growth factor receptor binding	11	0.407085
GO:0051239 regulation of multicellular organismal process	10	0.390475
GO:0015485 cholesterol binding	10	0.390309
GO:0007243 intracellular protein kinase cascade	30	0.38602
GO:0032259 methylation	27	0.385824

GO:0046907 intracellular transport	14	0.383958
GO:0016740 transferase activity	45	0.371321
GO:0031323 regulation of cellular metabolic process	12	0.369817
GO:0006906 vesicle fusion	10	0.369529
GO:0009314 response to radiation	13	0.361845
GO:0006631 fatty acid metabolic process	14	0.356433
GO:0034612 response to tumor necrosis factor	10	0.344071
GO:0001649 osteoblast differentiation	13	0.337755
GO:0003333 amino acid transmembrane transport	19	0.33351
GO:0005545 1-phosphatidylinositol binding	17	0.332585
GO:0004091 carboxylesterase activity	10	0.332194
GO:0009987 cellular process	85	0.329877
GO:0005547 phosphatidylinositol-3,4,5-trisphosphate binding	10	0.329318
GO:0002020 protease binding	19	0.327888
GO:0005802 trans-Golgi network	40	0.327285
GO:0005200 structural constituent of cytoskeleton	41	0.326731
GO:0043229 intracellular organelle	24	0.326059
GO:0005901 caveola	28	0.323656
GO:0030018 Z disc	48	0.317311
GO:0007169 transmembrane receptor protein tyrosine kinase signaling pathway	15	0.316309
GO:0032501 multicellular organismal process	33	0.315918
GO:0008629 induction of apoptosis by intracellular signals	16	0.312874
GO:0004674 protein serine/threonine kinase activity	116	0.309064
GO:0004252 serine-type endopeptidase activity	34	0.308873
GO:0045773 positive regulation of axon extension	10	0.306196
GO:0006954 inflammatory response	44	0.305871
GO:0005576 extracellular region	158	0.304464
GO:0048523 negative regulation of cellular process	15	0.303374
GO:0006468 protein phosphorylation	109	0.303163
GO:0043154 negative regulation of cysteine-type endopeptidase activity involved in apoptotic process	17	0.301995
GO:0050896 response to stimulus	40	0.300552
GO:0019899 enzyme binding	77	0.297405
GO:0031981 nuclear lumen	11	0.297224
GO:0016323 basolateral plasma membrane	47	0.29565
GO:0031410 cytoplasmic vesicle	34	0.292915
GO:0042254 ribosome biogenesis	21	0.292106
GO:0005544 calcium-dependent phospholipid binding	11	0.29163
GO:0002376 immune system process	12	0.290465
GO:0007269 neurotransmitter secretion	18	0.289863
GO:0016324 apical plasma membrane	62	0.288346
GO:0044430 cytoskeletal part	20	0.288183
GO:0022625 cytosolic large ribosomal subunit	16	0.287556

GO:0044446 intracellular organelle part	47	0.285464
GO:0006805 xenobiotic metabolic process	58	0.28443
GO:0000187 activation of MAPK activity	28	0.282872
GO:0043234 protein complex	100	0.281958
GO:0006956 complement activation	10	0.281426
GO:0005777 peroxisome	23	0.279195
GO:0030049 muscle filament sliding	18	0.276684
GO:0006917 induction of apoptosis	66	0.276162
GO:0008093 cytoskeletal adaptor activity	16	0.275868
GO:0009653 anatomical structure morphogenesis	30	0.275373
GO:0035085 cilium axoneme	15	0.275291
GO:0003824 catalytic activity	24	0.274538
GO:0008201 heparin binding	37	0.272998
GO:0008092 cytoskeletal protein binding	21	0.272721
GO:0048519 negative regulation of biological process	11	0.27157
GO:0050794 regulation of cellular process	49	0.271512
GO:0044424 intracellular part	66	0.270764
GO:0000118 histone deacetylase complex	11	0.269944
GO:0006811 ion transport	26	0.269625
GO:0044260 cellular macromolecule metabolic process	17	0.268881
GO:0007204 elevation of cytosolic calcium ion concentration	12	0.268599
GO:0008217 regulation of blood pressure	19	0.267875
GO:0006921 cellular component disassembly involved in apoptotic process	32	0.267356
GO:0006259 DNA metabolic process	13	0.266688
GO:0044267 cellular protein metabolic process	14	0.265348
GO:0043231 intracellular membrane-bounded organelle	67	0.264188
GO:0044237 cellular metabolic process	17	0.263895
GO:0030509 BMP signaling pathway	15	0.262512
GO:0071363 cellular response to growth factor stimulus	10	0.261966
GO:0050789 regulation of biological process	15	0.259785
GO:0045740 positive regulation of DNA replication	12	0.259583
GO:0031966 mitochondrial membrane	20	0.258731
GO:0005815 microtubule organizing center	39	0.2577
GO:0007219 Notch signaling pathway	12	0.257301
GO:0006508 proteolysis	100	0.255734
GO:0048856 anatomical structure development	13	0.255674
GO:0043410 positive regulation of MAPK cascade	22	0.254811
GO:0007186 G-protein coupled receptor signaling pathway	33	0.252363
GO:0006488 dolichol-linked oligosaccharide biosynthetic process	17	0.252347
GO:0008203 cholesterol metabolic process	24	0.252137
GO:0005516 calmodulin binding	58	0.25201
GO:0006974 response to DNA damage stimulus	31	0.251939

GO:0010629 negative regulation of gene expression	15	0.251842
GO:0004197 cysteine-type endopeptidase activity	29	0.250603
GO:0006486 protein glycosylation	14	0.244156
GO:0045454 cell redox homeostasis	29	0.243823
GO:0009636 response to toxin	36	0.243525
GO:0019825 oxygen binding	19	0.243498
GO:0030659 cytoplasmic vesicle membrane	22	0.242149
GO:0005813 centrosome	101	0.241051
GO:0009615 response to virus	33	0.240405
GO:0032868 response to insulin stimulus	18	0.239624
GO:0015758 glucose transport	10	0.239532
GO:0005198 structural molecule activity	62	0.238431
GO:0048471 perinuclear region of cytoplasm	197	0.237909
GO:0043548 phosphatidylinositol 3-kinase binding	11	0.237721
GO:0016787 hydrolase activity	60	0.237539
GO:0044464 cell part	42	0.233987
GO:0044425 membrane part	37	0.233707
GO:0007030 Golgi organization	16	0.231388
GO:0033574 response to testosterone stimulus	11	0.231313
GO:0045087 innate immune response	29	0.231229
GO:0042221 response to chemical stimulus	15	0.230532
GO:0032502 developmental process	25	0.230198
GO:0001669 acrosomal vesicle	16	0.229535
GO:0007605 sensory perception of sound	34	0.229375
GO:0001701 in utero embryonic development	89	0.229115
GO:0005488 binding	211	0.228708
GO:0016043 cellular component organization	16	0.228119
GO:0005814 centriole	15	0.22721
GO:0042632 cholesterol homeostasis	26	0.226171
GO:0031012 extracellular matrix	25	0.226078
GO:0032266 phosphatidylinositol-3-phosphate binding	10	0.225818
GO:0003007 heart morphogenesis	16	0.225487
GO:0001501 skeletal system development	33	0.225114
GO:0006641 triglyceride metabolic process	10	0.224709
GO:0018108 peptidyl-tyrosine phosphorylation	13	0.224574
GO:0007052 mitotic spindle organization	14	0.224518
GO:0006120 mitochondrial electron transport, NADH to ubiquinone	16	0.223952
GO:0010332 response to gamma radiation	10	0.223909
GO:0007283 spermatogenesis	101	0.222994
GO:0031295 T cell costimulation	18	0.221782
GO:0000086 G2/M transition of mitotic cell cycle	65	0.221589
GO:0006887 exocytosis	21	0.2214

GO:0007173 epidermal growth factor receptor signaling pathway	29	0.220091
GO:0005829 cytosol	1124	0.219816
GO:0003730 mRNA 3'-UTR binding	21	0.21939
GO:0019915 lipid storage	10	0.218391
GO:0007040 lysosome organization	14	0.218376
GO:0008233 peptidase activity	15	0.217541
GO:0009060 aerobic respiration	10	0.217507
GO:0006874 cellular calcium ion homeostasis	22	0.215547
GO:0031593 polyubiquitin binding	12	0.215417
GO:0035091 phosphatidylinositol binding	21	0.215136
GO:0006351 transcription, DNA-dependent	69	0.21431
GO:0004518 nuclease activity	10	0.214279
GO:0017111 nucleoside-triphosphatase activity	23	0.21392
GO:0046326 positive regulation of glucose import	13	0.213804
GO:0008654 phospholipid biosynthetic process	14	0.213772
GO:0000062 fatty-acyl-CoA binding	14	0.213744
GO:0000792 heterochromatin	17	0.213428
GO:0060027 convergent extension involved in gastrulation	10	0.213358
GO:0007165 signal transduction	151	0.213111
GO:0071260 cellular response to mechanical stimulus	10	0.212449
GO:0006694 steroid biosynthetic process	10	0.210955
GO:0001822 kidney development	32	0.209622
GO:0016020 membrane	230	0.208668
GO:0007584 response to nutrient	27	0.208645
GO:0048538 thymus development	12	0.208474
GO:0005747 mitochondrial respiratory chain complex I	22	0.20709
GO:0008137 NADH dehydrogenase (ubiquinone) activity	18	0.207042
GO:0048477 oogenesis	11	0.205838
GO:0048260 positive regulation of receptor-mediated endocytosis	13	0.205697
GO:0000236 mitotic prometaphase	44	0.205216
GO:0006521 regulation of cellular amino acid metabolic process	35	0.204565
GO:0006302 double-strand break repair	24	0.204233
GO:0003725 double-stranded RNA binding	19	0.204218
GO:0001533 cornified envelope	10	0.204082
GO:0006913 nucleocytoplasmic transport	18	0.203272
GO:0031100 organ regeneration	24	0.201765
GO:0045787 positive regulation of cell cycle	14	0.201341
GO:0007596 blood coagulation	98	0.200416
GO:0005506 iron ion binding	34	0.198198
GO:0022900 electron transport chain	14	0.197126
GO:0022904 respiratory electron transport chain	15	0.197022
GO:0004888 transmembrane signaling receptor activity	10	0.196433

GO:0065008 regulation of biological quality	11	0.195385
GO:0005739 mitochondrion	401	0.195155
GO:0016712 oxidoreductase activity, acting on paired donors, with incorporation or reduction of molecular oxygen, reduced flavin or flavoprotein as one donor, and incorporation of one atom of oxygen	10	0.195036
GO:0005875 microtubule associated complex	18	0.193707
GO:0006699 bile acid biosynthetic process	13	0.19296
GO:0030100 regulation of endocytosis	14	0.192939
GO:0042493 response to drug	126	0.192654
GO:0014823 response to activity	10	0.192199
GO:0048487 beta-tubulin binding	16	0.192016
GO:0004497 monooxygenase activity	10	0.191217
GO:0009950 dorsal/ventral axis specification	11	0.190771
GO:0016853 isomerase activity	11	0.188143
GO:0005515 protein binding	1796	0.187994
GO:0005737 cytoplasm	855	0.187333
GO:0071842 cellular component organization at cellular level	11	0.187008
GO:0043524 negative regulation of neuron apoptotic process	37	0.186927
GO:0043015 gamma-tubulin binding	10	0.186857
GO:0005730 nucleolus	668	0.185626
GO:0003676 nucleic acid binding	124	0.185326
GO:0051897 positive regulation of protein kinase B signaling cascade	19	0.185076
GO:0030122 AP-2 adaptor complex	10	0.185
GO:0008152 metabolic process	61	0.184949
GO:0005622 intracellular	74	0.184909
GO:0044444 cytoplasmic part	81	0.184531
GO:0031965 nuclear membrane	59	0.184318
GO:0004867 serine-type endopeptidase inhibitor activity	15	0.184296
GO:0006464 cellular protein modification process	24	0.183515
GO:0006810 transport	97	0.183133
GO:0045860 positive regulation of protein kinase activity	10	0.181886
GO:0045892 negative regulation of transcription, DNA-dependent	118	0.181827
GO:0008168 methyltransferase activity	21	0.181762
GO:0055114 oxidation-reduction process	186	0.18157
GO:0031090 organelle membrane	33	0.180666
GO:0007154 cell communication	24	0.180628
GO:0005624 membrane fraction	180	0.180096
GO:0006284 base-excision repair	13	0.179959
GO:0005792 microsome	152	0.178133
GO:0006520 cellular amino acid metabolic process	16	0.177307
GO:0050852 T cell receptor signaling pathway	26	0.176999

GO:0045095 keratin filament	10	0.176145
GO:0006996 organelle organization	24	0.175634
GO:0031901 early endosome membrane	29	0.175021
GO:0008144 drug binding	26	0.17438
GO:0003690 double-stranded DNA binding	50	0.173584
GO:0042645 mitochondrial nucleoid	26	0.173241
GO:0005615 extracellular space	163	0.173098
GO:0005764 lysosome	75	0.172159
GO:0005507 copper ion binding	18	0.171754
GO:0007517 muscle organ development	35	0.17151
GO:0009055 electron carrier activity	65	0.171114
GO:0010468 regulation of gene expression	16	0.170592
GO:0015035 protein disulfide oxidoreductase activity	12	0.170146
GO:0043232 intracellular non-membrane-bounded organelle	14	0.169794
GO:0007623 circadian rhythm	16	0.169712
GO:0048589 developmental growth	12	0.168114
GO:0005178 integrin binding	39	0.167786
GO:0005789 endoplasmic reticulum membrane	216	0.167574
GO:0016567 protein ubiquitination	73	0.167569
GO:0000776 kinetochore	27	0.167515
GO:0016581 NuRD complex	16	0.167465
GO:0030173 integral to Golgi membrane	14	0.166814
GO:0016477 cell migration	37	0.166068
GO:0042169 SH2 domain binding	24	0.166041
GO:0000278 mitotic cell cycle	17	0.164239
GO:0032088 negative regulation of NF-kappaB transcription factor activity	22	0.164201
GO:0007131 reciprocal meiotic recombination	15	0.163931
GO:0006935 chemotaxis	11	0.163763
GO:0006916 anti-apoptosis	85	0.163649
GO:0000045 autophagic vacuole assembly	12	0.163233
GO:0019897 extrinsic to plasma membrane	11	0.163091
GO:0005070 SH3/SH2 adaptor activity	12	0.162417
GO:0042803 protein homodimerization activity	237	0.162262
GO:0007163 establishment or maintenance of cell polarity	17	0.161845
GO:0050790 regulation of catalytic activity	33	0.161809
GO:0043010 camera-type eye development	11	0.161405
GO:0046676 negative regulation of insulin secretion	10	0.161018
GO:0008284 positive regulation of cell proliferation	108	0.161001
GO:0020037 heme binding	36	0.160872
GO:0009925 basal plasma membrane	11	0.160841
GO:0001947 heart looping	12	0.159983
GO:0004386 helicase activity	21	0.159901
GO:0003723 RNA binding	218	0.159437

GO:0040011 locomotion	18	0.159409
GO:0007626 locomotory behavior	21	0.158397
GO:0043169 cation binding	10	0.157775
GO:0031225 anchored to membrane	10	0.157508
GO:0016604 nuclear body	12	0.157487
GO:0051789 response to protein stimulus	14	0.15724
GO:0046872 metal ion binding	376	0.156785
GO:0045296 cadherin binding	14	0.156502
GO:0030016 myofibril	10	0.156479
GO:0042325 regulation of phosphorylation	13	0.155781
GO:0007528 neuromuscular junction development	15	0.155418
GO:0046677 response to antibiotic	13	0.155337
GO:0071222 cellular response to lipopolysaccharide	14	0.155229
GO:0042995 cell projection	37	0.155055
GO:0065007 biological regulation	12	0.154772
GO:0045111 intermediate filament cytoskeleton	28	0.154616
GO:0048255 mRNA stabilization	11	0.154489
GO:0012505 endomembrane system	54	0.154129
GO:0005765 lysosomal membrane	39	0.153237
GO:0007018 microtubule-based movement	36	0.152718
GO:0032355 response to estradiol stimulus	36	0.152639
GO:0001890 placenta development	10	0.152551
GO:0031572 G2/M transition DNA damage checkpoint	13	0.152428
GO:0042157 lipoprotein metabolic process	10	0.152305
GO:0090304 nucleic acid metabolic process	10	0.152273
GO:0007049 cell cycle	85	0.152172
GO:0000932 cytoplasmic mRNA processing body	23	0.151934
GO:0042542 response to hydrogen peroxide	20	0.151077
GO:0007005 mitochondrion organization	11	0.15105
GO:0009967 positive regulation of signal transduction	12	0.150872
GO:0042552 myelination	12	0.149968
GO:0016197 endosomal transport	27	0.149843
GO:0005886 plasma membrane	475	0.149613
GO:0048705 skeletal system morphogenesis	10	0.149557
GO:0007155 cell adhesion	61	0.149476
GO:0043401 steroid hormone mediated signaling pathway	16	0.148889
GO:0005158 insulin receptor binding	19	0.148358
GO:0006952 defense response	24	0.147479
GO:0005887 integral to plasma membrane	197	0.147339
GO:0003697 single-stranded DNA binding	41	0.14685
GO:0005102 receptor binding	61	0.146845
GO:0051216 cartilage development	13	0.146292
GO:0000242 pericentriolar material	11	0.14624
GO:0016023 cytoplasmic membrane-bounded vesicle	50	0.145745

GO:0005671 Ada2/Gcn5/Ada3 transcription activator complex	10	0.145679
GO:0042102 positive regulation of T cell proliferation	11	0.145259
GO:0005769 early endosome	64	0.145214
GO:0010951 negative regulation of endopeptidase activity	16	0.145136
GO:0005759 mitochondrial matrix	106	0.144474
GO:0007409 axonogenesis	19	0.143723
GO:0016235 aggresome	10	0.143722
GO:0018149 peptide cross-linking	11	0.143424
GO:0071339 MLL1 complex	25	0.143412
GO:0007010 cytoskeleton organization	14	0.143255
GO:0006986 response to unfolded protein	24	0.143133
GO:0008543 fibroblast growth factor receptor signaling pathway	30	0.142889
GO:0017137 Rab GTPase binding	15	0.142445
GO:0008283 cell proliferation	117	0.142321
GO:0001556 oocyte maturation	10	0.141729
GO:0030424 axon	50	0.141657
GO:0030511 positive regulation of transforming growth factor beta receptor signaling pathway	11	0.141501
GO:0001824 blastocyst development	10	0.14123
GO:0051225 spindle assembly	15	0.140958
GO:0007411 axon guidance	115	0.140612
GO:0016192 vesicle-mediated transport	52	0.140187
GO:0043627 response to estrogen stimulus	27	0.140119
GO:0048522 positive regulation of cellular process	33	0.140046
GO:0043433 negative regulation of sequence-specific DNA binding transcription factor activity	23	0.139907
GO:0005044 scavenger receptor activity	10	0.139675
GO:0030163 protein catabolic process	11	0.139565
GO:0030198 extracellular matrix organization	23	0.139387
GO:0048518 positive regulation of biological process	10	0.139013
GO:0045444 fat cell differentiation	19	0.138963
GO:0001558 regulation of cell growth	22	0.138376
GO:0005635 nuclear envelope	33	0.137938
GO:0005856 cytoskeleton	122	0.137585
GO:0050660 flavin adenine dinucleotide binding	42	0.137427
GO:0007399 nervous system development	64	0.137248
GO:0006396 RNA processing	24	0.136031
GO:0001568 blood vessel development	16	0.135929
GO:0035064 methylated histone residue binding	26	0.135927
GO:0030426 growth cone	45	0.135923
GO:0008083 growth factor activity	25	0.135852
GO:0032092 positive regulation of protein binding	19	0.135792
GO:0008023 transcription elongation factor complex	11	0.135721
GO:0007389 pattern specification process	11	0.135571

GO:0030183 B cell differentiation	11	0.13537
GO:0050885 neuromuscular process controlling balance	16	0.135128
GO:0042802 identical protein binding	196	0.135032
GO:0006955 immune response	45	0.134941
GO:0008017 microtubule binding	41	0.134576
GO:0048146 positive regulation of fibroblast proliferation	15	0.133805
GO:0017124 SH3 domain binding	54	0.133721
GO:0001658 branching involved in ureteric bud morphogenesis	13	0.133526
GO:0043204 perikaryon	12	0.13345
GO:0008286 insulin receptor signaling pathway	78	0.133116
GO:0008415 transferase activity, transferring acyl groups	13	0.133084
GO:0072593 reactive oxygen species metabolic process	11	0.132958
GO:0046329 negative regulation of JNK cascade	11	0.132833
GO:0019903 protein phosphatase binding	27	0.132799
GO:0001934 positive regulation of protein phosphorylation	28	0.132657
GO:0007257 activation of JUN kinase activity	13	0.132574
GO:0032403 protein complex binding	94	0.132527
GO:0008624 induction of apoptosis by extracellular signals	44	0.132524
GO:0006897 endocytosis	57	0.132488
GO:0030512 negative regulation of transforming growth factor beta receptor signaling pathway	15	0.132453
GO:0007126 meiosis	21	0.132344
GO:0001933 negative regulation of protein phosphorylation	15	0.132206
GO:0005634 nucleus	980	0.131948
GO:0003777 microtubule motor activity	25	0.131732
GO:0051384 response to glucocorticoid stimulus	35	0.131651
GO:0000166 nucleotide binding	186	0.131555
GO:0008094 DNA-dependent ATPase activity	23	0.130972
GO:0008234 cysteine-type peptidase activity	17	0.130968
GO:0030335 positive regulation of cell migration	46	0.130591
GO:0004519 endonuclease activity	14	0.13026
GO:0032091 negative regulation of protein binding	12	0.130042
GO:0080008 CUL4-RING ubiquitin ligase complex	11	0.129837
GO:0043161 proteasomal ubiquitin-dependent protein catabolic process	24	0.129541
GO:0030528 transcription regulator activity	37	0.129471
GO:0008104 protein localization	30	0.129356
GO:0007254 JNK cascade	13	0.129122
GO:0019233 sensory perception of pain	11	0.129088
GO:0009306 protein secretion	11	0.129059
GO:0014070 response to organic cyclic compound	54	0.129017
GO:0000718 nucleotide-excision repair, DNA damage removal	16	0.128978
GO:0000299 integral to membrane of membrane fraction	14	0.128551

GO:0043124 negative regulation of I-kappaB kinase/NF-kappaB cascade	10	0.128538
GO:0007507 heart development	45	0.128506
GO:0045768 positive regulation of anti-apoptosis	16	0.128432
GO:0006281 DNA repair	81	0.128349
GO:0005529 carbohydrate binding	23	0.128328
GO:0008143 poly(A) RNA binding	16	0.127623
GO:0033077 T cell differentiation in thymus	14	0.127463
GO:0060070 canonical Wnt receptor signaling pathway	15	0.127413
GO:0007166 cell surface receptor signaling pathway	30	0.127348
GO:0042594 response to starvation	12	0.127178
GO:0031902 late endosome membrane	29	0.12716
GO:0070064 proline-rich region binding	10	0.127124
GO:0005876 spindle microtubule	13	0.126931
GO:0040035 hermaphrodite genitalia development	13	0.126683
GO:0016575 histone deacetylation	10	0.12655
GO:0031175 neuron projection development	25	0.126269
GO:0005932 microtubule basal body	16	0.126109
GO:0009986 cell surface	93	0.126031
GO:0005654 nucleoplasm	345	0.125747
GO:0005643 nuclear pore	34	0.125729
GO:0048015 phosphatidylinositol-mediated signaling	35	0.125661
GO:0034394 protein localization at cell surface	13	0.125225
GO:0016042 lipid catabolic process	12	0.125138
GO:0007156 homophilic cell adhesion	16	0.125125
GO:0008565 protein transporter activity	40	0.124938
GO:0034339 regulation of transcription from RNA polymerase II promoter by nuclear hormone receptor	22	0.124684
GO:0042393 histone binding	31	0.124631
GO:0006364 rRNA processing	40	0.124093
GO:0051865 protein autoubiquitination	18	0.124037
GO:0009790 embryo development	49	0.12363
GO:0051028 mRNA transport	28	0.12319
GO:0007568 aging	43	0.122862
GO:0051289 protein homotetramerization	18	0.122679
GO:0030308 negative regulation of cell growth	51	0.122532
GO:0051539 4 iron, 4 sulfur cluster binding	13	0.122435
GO:0006412 translation	44	0.122162
GO:0019717 synaptosome	46	0.121632
GO:0006310 DNA recombination	18	0.121495
GO:0005795 Golgi stack	16	0.121363
GO:0005768 endosome	46	0.121221
GO:0042593 glucose homeostasis	31	0.121077
GO:0021987 cerebral cortex development	13	0.12072

GO:0043065 positive regulation of apoptotic process	67	0.120621
GO:0018279 protein N-linked glycosylation via asparagine	57	0.120617
GO:0004930 G-protein coupled receptor activity	14	0.120213
GO:0055010 ventricular cardiac muscle tissue morphogenesis	13	0.120174
GO:0050821 protein stabilization	30	0.119525
GO:0009411 response to UV	13	0.11934
GO:0042626 ATPase activity, coupled to transmembrane movement of substances	11	0.119245
GO:0016887 ATPase activity	44	0.119175
GO:0006814 sodium ion transport	27	0.119046
GO:0005758 mitochondrial intermembrane space	21	0.118965
GO:0008022 protein C-terminus binding	97	0.118915
GO:0051056 regulation of small GTPase mediated signal transduction	52	0.118739
GO:0005096 GTPase activator activity	48	0.1187
GO:0005783 endoplasmic reticulum	188	0.118616
GO:0006944 cellular membrane fusion	15	0.118474
GO:0000289 nuclear-transcribed mRNA poly(A) tail shortening	19	0.11816
GO:0032496 response to lipopolysaccharide	36	0.117892
GO:0016702 oxidoreductase activity, acting on single donors with incorporation of molecular oxygen, incorporation of two atoms of oxygen	21	0.117715
GO:0006879 cellular iron ion homeostasis	36	0.117611
GO:0005844 polysome	13	0.117366
GO:0030331 estrogen receptor binding	14	0.117334
GO:0040014 regulation of multicellular organism growth	13	0.11723
GO:0030170 pyridoxal phosphate binding	29	0.11702
GO:0042470 melanosome	58	0.116872
GO:0017147 Wnt-protein binding	10	0.116844
GO:0070301 cellular response to hydrogen peroxide	13	0.116564
GO:0019221 cytokine-mediated signaling pathway	20	0.11645
GO:0006629 lipid metabolic process	29	0.116443
GO:0000910 cytokinesis	29	0.116431
GO:0005089 Rho guanyl-nucleotide exchange factor activity	23	0.116184
GO:0007417 central nervous system development	34	0.115772
GO:0007420 brain development	57	0.115713
GO:0051059 NF-kappaB binding	16	0.115642
GO:0030529 ribonucleoprotein complex	45	0.115572
GO:0001666 response to hypoxia	75	0.115329
GO:0000724 double-strand break repair via homologous recombination	21	0.115322
GO:0045739 positive regulation of DNA repair	13	0.115295
GO:0006091 generation of precursor metabolites and energy	16	0.115249
GO:0045727 positive regulation of translation	24	0.115148

GO:0008047 enzyme activator activity	15	0.114822
GO:0001702 gastrulation with mouth forming second	15	0.114761
GO:0007369 gastrulation	15	0.114597
GO:0005743 mitochondrial inner membrane	128	0.114557
GO:0043531 ADP binding	16	0.114265
GO:0000902 cell morphogenesis	23	0.114023
GO:0016301 kinase activity	21	0.113986
GO:0042981 regulation of apoptotic process	63	0.113904
GO:0006270 DNA-dependent DNA replication initiation	13	0.113758
GO:0043014 alpha-tubulin binding	12	0.113576
GO:0042384 cilium assembly	20	0.113573
GO:0016021 integral to membrane	645	0.113481
GO:0060041 retina development in camera-type eye	23	0.113082
GO:0004721 phosphoprotein phosphatase activity	16	0.112978
GO:0045211 postsynaptic membrane	35	0.112951
GO:0045202 synapse	33	0.112807
GO:0006665 sphingolipid metabolic process	11	0.112697
GO:0051117 ATPase binding	12	0.112681
GO:0007067 mitosis	76	0.112636
GO:0035556 intracellular signal transduction	60	0.112437
GO:0009303 rRNA transcription	10	0.112419
GO:0045669 positive regulation of osteoblast differentiation	20	0.112116
GO:0001525 angiogenesis	50	0.111649
GO:0000922 spindle pole	36	0.111642
GO:0060325 face morphogenesis	13	0.111533
GO:0045732 positive regulation of protein catabolic process	17	0.11153
GO:0050661 NADP binding	20	0.111202
GO:0007059 chromosome segregation	30	0.110379
GO:0051262 protein tetramerization	14	0.110371
GO:0043066 negative regulation of apoptotic process	95	0.110309
GO:0001657 ureteric bud development	10	0.110272
GO:0007498 mesoderm development	13	0.110169
GO:0015293 symporter activity	10	0.109929
GO:0060334 regulation of interferon-gamma-mediated signaling pathway	10	0.109896
GO:0008021 synaptic vesicle	30	0.109802
GO:0043197 dendritic spine	29	0.109492
GO:0008013 beta-catenin binding	42	0.109462
GO:0014069 postsynaptic density	33	0.109301
GO:0003714 transcription corepressor activity	89	0.109282
GO:0003707 steroid hormone receptor activity	13	0.109081
GO:0016525 negative regulation of angiogenesis	13	0.109015
GO:0004871 signal transducer activity	94	0.108932
GO:0005100 Rho GTPase activator activity	10	0.108775

GO:0031398 positive regulation of protein ubiquitination	18	0.108752
GO:0045471 response to ethanol	33	0.108578
GO:0009952 anterior/posterior pattern specification	30	0.108497
GO:0008270 zinc ion binding	441	0.108401
GO:0048008 platelet-derived growth factor receptor signaling pathway	12	0.108338
GO:0050679 positive regulation of epithelial cell proliferation	14	0.108308
GO:0006144 purine nucleobase metabolic process	14	0.108279
GO:0010008 endosome membrane	54	0.108238
GO:0005788 endoplasmic reticulum lumen	48	0.108012
GO:0019432 triglyceride biosynthetic process	18	0.107906
GO:0045736 negative regulation of cyclin-dependent protein kinase activity	10	0.107904
GO:0030176 integral to endoplasmic reticulum membrane	30	0.10785
GO:0050772 positive regulation of axonogenesis	17	0.107823
GO:0006501 C-terminal protein lipidation	13	0.107626
GO:0030139 endocytic vesicle	12	0.107576
GO:0007595 lactation	20	0.107471
GO:0031093 platelet alpha granule lumen	16	0.107434
GO:0008360 regulation of cell shape	33	0.10639
GO:0016922 ligand-dependent nuclear receptor binding	14	0.106356
GO:0030159 receptor signaling complex scaffold activity	10	0.106347
GO:0030216 keratinocyte differentiation	22	0.106202
GO:0043130 ubiquitin binding	16	0.10619
GO:0006606 protein import into nucleus	24	0.105783
GO:0016049 cell growth	28	0.105761
GO:0016010 dystrophin-associated glycoprotein complex	11	0.105527
GO:0007368 determination of left/right symmetry	15	0.105367
GO:0046627 negative regulation of insulin receptor signaling pathway	11	0.105269
GO:0043547 positive regulation of GTPase activity	53	0.105243
GO:0006816 calcium ion transport	15	0.104342
GO:0018105 peptidyl-serine phosphorylation	29	0.10428
GO:0001889 liver development	48	0.104262
GO:0043123 positive regulation of I-kappaB kinase/NF-kappaB cascade	52	0.104222
GO:0006605 protein targeting	22	0.104216
GO:0090090 negative regulation of canonical Wnt receptor signaling pathway	29	0.103994
GO:0016605 PML body	42	0.103838
GO:0051260 protein homooligomerization	41	0.103802
GO:0048009 insulin-like growth factor receptor signaling pathway	11	0.103731
GO:0006950 response to stress	35	0.103716

GO:0005770 late endosome	28	0.103694
GO:0042475 odontogenesis of dentin-containing tooth	17	0.103688
GO:0005912 adherens junction	10	0.103602
GO:0007032 endosome organization	16	0.103471
GO:0006783 heme biosynthetic process	12	0.103441
GO:0010033 response to organic substance	26	0.103275
GO:0006355 regulation of transcription, DNA-dependent	261	0.103131
GO:0006366 transcription from RNA polymerase II promoter	59	0.103046
GO:0010043 response to zinc ion	15	0.102956
GO:0016180 snRNA processing	14	0.102205
GO:0032039 integrator complex	14	0.102205
GO:0005975 carbohydrate metabolic process	36	0.102185
GO:0000077 DNA damage checkpoint	20	0.101938
GO:0006970 response to osmotic stress	10	0.101738
GO:0001968 fibronectin binding	10	0.1017
GO:0000122 negative regulation of transcription from RNA polymerase II promoter	161	0.101583
GO:0003682 chromatin binding	117	0.101526
GO:0004725 protein tyrosine phosphatase activity	22	0.101323
GO:0000790 nuclear chromatin	27	0.101308
GO:0031941 filamentous actin	21	0.101277
GO:0016491 oxidoreductase activity	37	0.100997
GO:0045893 positive regulation of transcription, DNA-dependent	128	0.100928
GO:0005689 U12-type spliceosomal complex	16	0.10074
GO:0006979 response to oxidative stress	60	0.100734
GO:0045944 positive regulation of transcription from RNA polymerase II promoter	187	0.100695
GO:0005694 chromosome	31	0.100668
GO:0019898 extrinsic to membrane	14	0.100646
GO:0046983 protein dimerization activity	23	0.100537
GO:0018107 peptidyl-threonine phosphorylation	13	0.100443
GO:0006297 nucleotide-excision repair, DNA gap filling	17	0.100067
GO:0046686 response to cadmium ion	15	0.099998
GO:0042733 embryonic digit morphogenesis	20	0.099995
GO:0001570 vasculogenesis	34	0.099883
GO:0051010 microtubule plus-end binding	13	0.099797
GO:0016568 chromatin modification	37	0.099743
GO:0003729 mRNA binding	34	0.099548
GO:0034332 adherens junction organization	13	0.099503
GO:0005905 coated pit	19	0.099276
GO:0055085 transmembrane transport	119	0.098823
GO:0006914 autophagy	13	0.098769
GO:0005625 soluble fraction	235	0.098591

GO:0030136 clathrin-coated vesicle	12	0.098562
GO:0003677 DNA binding	383	0.098464
GO:0005518 collagen binding	11	0.098394
GO:0007265 Ras protein signal transduction	26	0.09837
GO:0032040 small-subunit processome	13	0.098244
GO:0009267 cellular response to starvation	13	0.098131
GO:0006260 DNA replication	48	0.098072
GO:0009887 organ morphogenesis	36	0.098031
GO:0051082 unfolded protein binding	67	0.09768
GO:0006333 chromatin assembly or disassembly	17	0.097609
GO:0005834 heterotrimeric G-protein complex	10	0.097603
GO:0008237 metallopeptidase activity	12	0.097447
GO:0046982 protein heterodimerization activity	118	0.097414
GO:0043085 positive regulation of catalytic activity	27	0.097259
GO:0016874 ligase activity	25	0.0968
GO:0006928 cellular component movement	31	0.09674
GO:0019843 rRNA binding	12	0.096584
GO:0005741 mitochondrial outer membrane	37	0.096457
GO:0044255 cellular lipid metabolic process	35	0.09633
GO:0043086 negative regulation of catalytic activity	22	0.096279
GO:0000049 tRNA binding	20	0.096132
GO:0006461 protein complex assembly	45	0.096125
GO:0051291 protein heterooligomerization	33	0.096028
GO:0046685 response to arsenic-containing substance	12	0.095894
GO:0030054 cell junction	62	0.09589
GO:0047485 protein N-terminus binding	48	0.095742
GO:0051301 cell division	126	0.095647
GO:0007015 actin filament organization	15	0.095627
GO:0034220 ion transmembrane transport	24	0.095613
GO:0000082 G1/S transition of mitotic cell cycle	60	0.09552
GO:0030178 negative regulation of Wnt receptor signaling pathway	22	0.095059
GO:0000139 Golgi membrane	150	0.09473
GO:0030199 collagen fibril organization	14	0.094685
GO:0005667 transcription factor complex	90	0.094555
GO:0003678 DNA helicase activity	11	0.094487
GO:0005794 Golgi apparatus	227	0.094426
GO:0007601 visual perception	47	0.094366
GO:0007569 cell aging	15	0.094346
GO:0005840 ribosome	29	0.094059
GO:0034644 cellular response to UV	12	0.093959
GO:0007275 multicellular organismal development	111	0.093939
GO:0000075 cell cycle checkpoint	32	0.093856

GO:0000472 endonucleolytic cleavage to generate mature 5'-end of SSU-rRNA from (SSU-rRNA, 5.8S rRNA, LSU-rRNA)	10	0.093643
GO:0000480 endonucleolytic cleavage in 5'-ETS of tricistronic rRNA transcript (SSU-rRNA, 5.8S rRNA, LSU-rRNA)	10	0.093643
GO:0032321 positive regulation of Rho GTPase activity	13	0.093574
GO:0006915 apoptotic process	96	0.093562
GO:0051015 actin filament binding	45	0.093543
GO:0010976 positive regulation of neuron projection development	12	0.093507
GO:0008544 epidermis development	16	0.093458
GO:0032201 telomere maintenance via semi-conservative replication	19	0.093422
GO:0045766 positive regulation of angiogenesis	25	0.093408
GO:0004672 protein kinase activity	29	0.093243
GO:0009888 tissue development	13	0.092892
GO:0005923 tight junction	32	0.092753
GO:0048793 pronephros development	11	0.092706
GO:0005546 phosphatidylinositol-4,5-bisphosphate binding	11	0.092616
GO:0008138 protein tyrosine/serine/threonine phosphatase activity	15	0.092578
GO:0042734 presynaptic membrane	14	0.092578
GO:0071456 cellular response to hypoxia	15	0.092535
GO:0040007 growth	19	0.092174
GO:0045121 membrane raft	39	0.092064
GO:0019901 protein kinase binding	100	0.091828
GO:0035023 regulation of Rho protein signal transduction	14	0.091776
GO:0004222 metalloendopeptidase activity	23	0.091689
GO:0048706 embryonic skeletal system development	10	0.09166
GO:0043966 histone H3 acetylation	32	0.091632
GO:0090263 positive regulation of canonical Wnt receptor signaling pathway	21	0.091408
GO:0017022 myosin binding	14	0.091087
GO:0030514 negative regulation of BMP signaling pathway	14	0.091064
GO:0003684 damaged DNA binding	17	0.091059
GO:0046330 positive regulation of JNK cascade	13	0.090952
GO:0005543 phospholipid binding	24	0.090889
GO:0003774 motor activity	15	0.090825
GO:0043022 ribosome binding	18	0.090418
GO:0045931 positive regulation of mitotic cell cycle	13	0.090314
GO:0030182 neuron differentiation	18	0.090284
GO:0007266 Rho protein signal transduction	19	0.090193
GO:0045785 positive regulation of cell adhesion	11	0.090187
GO:0009897 external side of plasma membrane	24	0.090171
GO:0005874 microtubule	81	0.08998

GO:0010827 regulation of glucose transport	18	0.089968
GO:0006470 protein dephosphorylation	30	0.089899
GO:0004872 receptor activity	69	0.089724
GO:0034605 cellular response to heat	10	0.089679
GO:0010628 positive regulation of gene expression	18	0.089662
GO:0040010 positive regulation of growth rate	23	0.089644
GO:0000722 telomere maintenance via recombination	20	0.089639
GO:0005604 basement membrane	25	0.089413
GO:0060766 negative regulation of androgen receptor signaling pathway	10	0.089238
GO:0008076 voltage-gated potassium channel complex	12	0.089151
GO:0030218 erythrocyte differentiation	18	0.08881
GO:0000387 spliceosomal snRNP assembly	14	0.088743
GO:0008285 negative regulation of cell proliferation	120	0.088722
GO:0043005 neuron projection	33	0.088709
GO:0001726 ruffle	39	0.088634
GO:0002053 positive regulation of mesenchymal cell proliferation	12	0.088544
GO:0006099 tricarboxylic acid cycle	20	0.088427
GO:0009612 response to mechanical stimulus	27	0.088402
GO:0045786 negative regulation of cell cycle	25	0.088018
GO:0043588 skin development	22	0.087997
GO:0050681 androgen receptor binding	34	0.087985
GO:0048286 lung alveolus development	13	0.087858
GO:0030036 actin cytoskeleton organization	53	0.087796
GO:0060395 SMAD protein signal transduction	10	0.087733
GO:0001755 neural crest cell migration	11	0.087703
GO:0035259 glucocorticoid receptor binding	10	0.087669
GO:0046961 proton-transporting ATPase activity, rotational mechanism	11	0.087566
GO:0006006 glucose metabolic process	20	0.087501
GO:0050731 positive regulation of peptidyl-tyrosine phosphorylation	14	0.087458
GO:0000775 chromosome, centromeric region	23	0.087456
GO:0031146 SCF-dependent proteasomal ubiquitin-dependent protein catabolic process	11	0.087456
GO:0051149 positive regulation of muscle cell differentiation	16	0.087431
GO:0000080 G1 phase of mitotic cell cycle	15	0.087426
GO:0000785 chromatin	45	0.087297
GO:0030971 receptor tyrosine kinase binding	14	0.087215
GO:0005811 lipid particle	10	0.087076
GO:0035264 multicellular organism growth	24	0.086986
GO:0030334 regulation of cell migration	16	0.086926
GO:0001503 ossification	22	0.086669

GO:0055037 recycling endosome	15	0.086587
GO:0050775 positive regulation of dendrite morphogenesis	11	0.086479
GO:0042127 regulation of cell proliferation	36	0.086402
GO:0005884 actin filament	19	0.086002
GO:0008134 transcription factor binding	116	0.085919
GO:0009749 response to glucose stimulus	33	0.085904
GO:0003713 transcription coactivator activity	115	0.085824
GO:0007346 regulation of mitotic cell cycle	13	0.08568
GO:0034329 cell junction assembly	21	0.085589
GO:0032580 Golgi cisterna membrane	17	0.084946
GO:0004722 protein serine/threonine phosphatase activity	19	0.084873
GO:0005125 cytokine activity	10	0.084872
GO:0060021 palate development	27	0.08487
GO:0043025 neuronal cell body	96	0.084841
GO:0006094 gluconeogenesis	25	0.084813
GO:0043434 response to peptide hormone stimulus	22	0.084632
GO:0006103 2-oxoglutarate metabolic process	14	0.084578
GO:0017053 transcriptional repressor complex	20	0.084381
GO:0030674 protein binding, bridging	34	0.084326
GO:0007565 female pregnancy	14	0.084162
GO:0048306 calcium-dependent protein binding	20	0.084158
GO:0008333 endosome to lysosome transport	21	0.084057
GO:0005578 proteinaceous extracellular matrix	31	0.084018
GO:0010862 positive regulation of pathway-restricted SMAD protein phosphorylation	11	0.083864
GO:0034097 response to cytokine stimulus	16	0.083762
GO:0015991 ATP hydrolysis coupled proton transport	14	0.083745
GO:0051726 regulation of cell cycle	39	0.083744
GO:0071565 nBAF complex	11	0.083653
GO:0006869 lipid transport	17	0.083625
GO:0016337 cell-cell adhesion	24	0.083395
GO:0008033 tRNA processing	13	0.083262
GO:0006635 fatty acid beta-oxidation	14	0.082969
GO:0030496 midbody	50	0.082542
GO:0042472 inner ear morphogenesis	15	0.08239
GO:0030864 cortical actin cytoskeleton	15	0.082382
GO:0030168 platelet activation	79	0.082381
GO:0030866 cortical actin cytoskeleton organization	10	0.082204
GO:0031124 mRNA 3'-end processing	18	0.081719
GO:0043967 histone H4 acetylation	16	0.081626
GO:0043499 eukaryotic cell surface binding	10	0.081621
GO:0009791 post-embryonic development	33	0.081523
GO:0051017 actin filament bundle assembly	15	0.081241
GO:0046854 phosphatidylinositol phosphorylation	15	0.081194

GO:0006271 DNA strand elongation involved in DNA replication	24	0.081172
GO:0040020 regulation of meiosis	11	0.080974
GO:0007566 embryo implantation	10	0.080941
GO:0007281 germ cell development	23	0.080904
GO:0019900 kinase binding	21	0.080868
GO:0003779 actin binding	104	0.080867
GO:0033572 transferrin transport	18	0.080855
GO:0002009 morphogenesis of an epithelium	11	0.080676
GO:0051591 response to cAMP	17	0.080506
GO:0002119 nematode larval development	31	0.080408
GO:0046716 muscle cell homeostasis	11	0.080326
GO:0032526 response to retinoic acid	23	0.080224
GO:0007094 mitotic cell cycle spindle assembly checkpoint	16	0.080195
GO:0007205 protein kinase C-activating G-protein coupled receptor signaling pathway	10	0.080121
GO:0007628 adult walking behavior	11	0.079512
GO:0003712 transcription cofactor activity	19	0.079293
GO:0006457 protein folding	73	0.079253
GO:0006898 receptor-mediated endocytosis	32	0.079141
GO:0016514 SWI/SNF complex	12	0.079032
GO:0015031 protein transport	161	0.079017
GO:0007267 cell-cell signaling	29	0.079009
GO:0004713 protein tyrosine kinase activity	12	0.079003
GO:0000447 endonucleolytic cleavage in ITS1 to separate SSU-rRNA from 5.8S rRNA and LSU-rRNA from tricistronic rRNA transcript (SSU-rRNA, 5.8S rRNA, LSU-rRNA)	13	0.078934
GO:0071230 cellular response to amino acid stimulus	11	0.078856
GO:0003899 DNA-directed RNA polymerase activity	20	0.078735
GO:0035116 embryonic hindlimb morphogenesis	15	0.078699
GO:0035335 peptidyl-tyrosine dephosphorylation	35	0.078584
GO:0051496 positive regulation of stress fiber assembly	12	0.078528
GO:0008219 cell death	33	0.078223
GO:0009725 response to hormone stimulus	24	0.078086
GO:0045295 gamma-catenin binding	10	0.07802
GO:0048661 positive regulation of smooth muscle cell proliferation	11	0.077912
GO:0030901 midbrain development	13	0.077617
GO:0031072 heat shock protein binding	32	0.077557
GO:0005782 peroxisomal matrix	25	0.077467
GO:0009953 dorsal/ventral pattern formation	11	0.077415
GO:0030154 cell differentiation	85	0.077376
GO:0006633 fatty acid biosynthetic process	11	0.077298
GO:0003887 DNA-directed DNA polymerase activity	13	0.077237

GO:0004177 aminopeptidase activity	14	0.077036
GO:0005201 extracellular matrix structural constituent	21	0.077035
GO:0042826 histone deacetylase binding	29	0.076725
GO:0030968 endoplasmic reticulum unfolded protein response	13	0.076724
GO:0008340 determination of adult lifespan	12	0.076662
GO:0007492 endoderm development	16	0.076613
GO:0004221 ubiquitin thiolesterase activity	35	0.0766
GO:0000216 M/G1 transition of mitotic cell cycle	59	0.076588
GO:0005085 guanyl-nucleotide exchange factor activity	26	0.076561
GO:0008289 lipid binding	28	0.076261
GO:0046777 protein autophosphorylation	49	0.075982
GO:0004843 ubiquitin-specific protease activity	29	0.075975
GO:0030155 regulation of cell adhesion	13	0.075933
GO:0000287 magnesium ion binding	95	0.075849
GO:0042446 hormone biosynthetic process	10	0.075802
GO:0007162 negative regulation of cell adhesion	15	0.075647
GO:0042326 negative regulation of phosphorylation	10	0.075301
GO:0016563 transcription activator activity	35	0.075216
GO:0007050 cell cycle arrest	54	0.075177
GO:0042623 ATPase activity, coupled	10	0.075167
GO:0071564 npBAF complex	12	0.075147
GO:0005097 Rab GTPase activator activity	26	0.075026
GO:0016328 lateral plasma membrane	18	0.075008
GO:0006378 mRNA polyadenylation	14	0.074873
GO:0030425 dendrite	52	0.07481
GO:0006406 mRNA export from nucleus	33	0.074755
GO:0032024 positive regulation of insulin secretion	13	0.074751
GO:0050900 leukocyte migration	43	0.074687
GO:0033613 activating transcription factor binding	11	0.074576
GO:0034446 substrate adhesion-dependent cell spreading	12	0.074454
GO:0048011 nerve growth factor receptor signaling pathway	106	0.074294
GO:0000184 nuclear-transcribed mRNA catabolic process, nonsense-mediated decay	19	0.074134
GO:0030742 GTP-dependent protein binding	10	0.074127
GO:0005109 frizzled binding	11	0.074048
GO:0030145 manganese ion binding	23	0.073722
GO:0000146 microfilament motor activity	11	0.073681
GO:0010212 response to ionizing radiation	22	0.073674
GO:0005913 cell-cell adherens junction	21	0.073607
GO:0030324 lung development	26	0.07346
GO:0048013 ephrin receptor signaling pathway	10	0.073453
GO:0008301 DNA binding, bending	12	0.073324
GO:0006413 translational initiation	27	0.073277
GO:0010811 positive regulation of cell-substrate adhesion	11	0.073208

GO:0046933 hydrogen ion transporting ATP synthase activity, rotational mechanism	10	0.073134
GO:0016607 nuclear speck	79	0.073029
GO:0005080 protein kinase C binding	20	0.072969
GO:0006357 regulation of transcription from RNA polymerase II promoter	100	0.072737
GO:0002039 p53 binding	31	0.072685
GO:0030141 secretory granule	19	0.072365
GO:0034641 cellular nitrogen compound metabolic process	12	0.072271
GO:0003705 RNA polymerase II distal enhancer sequence-specific DNA binding transcription factor activity	29	0.07222
GO:0009611 response to wounding	13	0.072212
GO:0000777 condensed chromosome kinetochore	21	0.071914
GO:0016032 viral reproduction	89	0.071833
GO:0046332 SMAD binding	20	0.071771
GO:0019005 SCF ubiquitin ligase complex	12	0.071684
GO:0005669 transcription factor TFIID complex	14	0.071682
GO:0035019 somatic stem cell maintenance	11	0.071651
GO:0015630 microtubule cytoskeleton	45	0.071381
GO:0042555 MCM complex	11	0.071096
GO:0005637 nuclear inner membrane	17	0.070945
GO:0006200 ATP catabolic process	97	0.070886
GO:0033138 positive regulation of peptidyl-serine phosphorylation	17	0.070838
GO:0000060 protein import into nucleus, translocation	11	0.070746
GO:0040008 regulation of growth	22	0.070526
GO:0000165 MAPK cascade	11	0.070513
GO:0007160 cell-matrix adhesion	22	0.070365
GO:0032154 cleavage furrow	12	0.070339
GO:0000413 protein peptidyl-prolyl isomerization	16	0.070296
GO:0030433 ER-associated protein catabolic process	24	0.070134
GO:0003700 sequence-specific DNA binding transcription factor activity	224	0.069884
GO:0005215 transporter activity	30	0.069766
GO:0043200 response to amino acid stimulus	18	0.069548
GO:0006309 apoptotic DNA fragmentation	10	0.069392
GO:0005938 cell cortex	53	0.069216
GO:0050771 negative regulation of axonogenesis	13	0.069131
GO:0005083 small GTPase regulator activity	13	0.069043
GO:0007585 respiratory gaseous exchange	11	0.068935
GO:0000151 ubiquitin ligase complex	32	0.0689
GO:0051219 phosphoprotein binding	13	0.06877
GO:0005626 insoluble fraction	21	0.06844
GO:0016310 phosphorylation	51	0.068386

GO:0033116 endoplasmic reticulum-Golgi intermediate compartment membrane	11	0.068333
GO:0002576 platelet degranulation	33	0.068186
GO:0001843 neural tube closure	27	0.068168
GO:0000084 S phase of mitotic cell cycle	80	0.068137
GO:0030521 androgen receptor signaling pathway	38	0.067977
GO:0032587 ruffle membrane	23	0.067842
GO:0043409 negative regulation of MAPK cascade	11	0.067807
GO:0032438 melanosome organization	11	0.067538
GO:0045862 positive regulation of proteolysis	10	0.067392
GO:0071445 cellular response to protein stimulus	13	0.066992
GO:0006754 ATP biosynthetic process	25	0.066456
GO:0043407 negative regulation of MAP kinase activity	17	0.066367
GO:0000209 protein polyubiquitination	21	0.066345
GO:0006112 energy reserve metabolic process	30	0.06633
GO:0030307 positive regulation of cell growth	28	0.066181
GO:0008380 RNA splicing	52	0.066173
GO:0071377 cellular response to glucagon stimulus	12	0.065995
GO:0045665 negative regulation of neuron differentiation	15	0.065941
GO:0010494 cytoplasmic stress granule	12	0.065755
GO:0015629 actin cytoskeleton	69	0.065586
GO:0070979 protein K11-linked ubiquitination	16	0.065514
GO:0007229 integrin-mediated signaling pathway	22	0.065447
GO:0006446 regulation of translational initiation	16	0.065445
GO:0001707 mesoderm formation	17	0.065295
GO:0005509 calcium ion binding	161	0.065138
GO:0009966 regulation of signal transduction	11	0.065095
GO:0006283 transcription-coupled nucleotide-excision repair	33	0.065062
GO:0030177 positive regulation of Wnt receptor signaling pathway	11	0.064983
GO:0009792 embryo development ending in birth or egg hatching	48	0.064831
GO:0046847 filopodium assembly	12	0.064721
GO:0019904 protein domain specific binding	115	0.064411
GO:0007519 skeletal muscle tissue development	27	0.064348
GO:0030217 T cell differentiation	12	0.064098
GO:0001764 neuron migration	25	0.06408
GO:0006361 transcription initiation from RNA polymerase I promoter	15	0.063975
GO:0005911 cell-cell junction	27	0.063564
GO:0032947 protein complex scaffold	15	0.063484
GO:0005819 spindle	43	0.063422
GO:0070830 tight junction assembly	13	0.063066
GO:0060048 cardiac muscle contraction	15	0.062996

GO:0001892 embryonic placenta development	11	0.062808
GO:0030027 lamellipodium	57	0.062743
GO:0006813 potassium ion transport	26	0.062597
GO:0030165 PDZ domain binding	23	0.062393
GO:0009898 internal side of plasma membrane	21	0.062162
GO:0006368 transcription elongation from RNA polymerase II promoter	48	0.062158
GO:0015030 Cajal body	23	0.062093
GO:0016363 nuclear matrix	40	0.061868
GO:0032436 positive regulation of proteasomal ubiquitin-dependent protein catabolic process	19	0.061852
GO:0016579 protein deubiquitination	17	0.061822
GO:0042787 protein ubiquitination involved in ubiquitin-dependent protein catabolic process	24	0.061754
GO:0070577 histone acetyl-lysine binding	11	0.061721
GO:0016597 amino acid binding	13	0.061469
GO:0003702 RNA polymerase II transcription factor activity	11	0.061402
GO:0016044 cellular membrane organization	38	0.061348
GO:0000083 regulation of transcription involved in G1/S phase of mitotic cell cycle	14	0.061321
GO:0044419 interspecies interaction between organisms	117	0.061273
GO:0016573 histone acetylation	15	0.061106
GO:0007179 transforming growth factor beta receptor signaling pathway	25	0.061038
GO:0032869 cellular response to insulin stimulus	23	0.060769
GO:0035267 NuA4 histone acetyltransferase complex	11	0.060718
GO:0001725 stress fiber	27	0.060628
GO:0032781 positive regulation of ATPase activity	11	0.060388
GO:0030097 hemopoiesis	23	0.060165
GO:0030863 cortical cytoskeleton	14	0.059966
GO:0051020 GTPase binding	11	0.059747
GO:0006821 chloride transport	15	0.059594
GO:0006891 intra-Golgi vesicle-mediated transport	16	0.059397
GO:0007286 spermatid development	15	0.059048
GO:0050699 WW domain binding	13	0.059014
GO:0043565 sequence-specific DNA binding	120	0.058826
GO:0050434 positive regulation of viral transcription	30	0.058493
GO:0042059 negative regulation of epidermal growth factor receptor signaling pathway	22	0.058439
GO:0016358 dendrite development	14	0.05826
GO:0005925 focal adhesion	65	0.058259
GO:0006367 transcription initiation from RNA polymerase II promoter	54	0.058183
GO:0006397 mRNA processing	61	0.057962

GO:0003755 peptidyl-prolyl cis-trans isomerase activity	21	0.057367
GO:0016071 mRNA metabolic process	46	0.057357
GO:0030336 negative regulation of cell migration	28	0.056812
GO:0005796 Golgi lumen	12	0.056528
GO:0006888 ER to Golgi vesicle-mediated transport	28	0.056527
GO:0006936 muscle contraction	32	0.056475
GO:0006417 regulation of translation	33	0.056167
GO:0031594 neuromuscular junction	15	0.056152
GO:0006362 transcription elongation from RNA polymerase I promoter	11	0.055931
GO:0008026 ATP-dependent helicase activity	29	0.055824
GO:0006338 chromatin remodeling	26	0.055372
GO:0005385 zinc ion transmembrane transporter activity	11	0.055308
GO:0000398 nuclear mRNA splicing, via spliceosome	94	0.055164
GO:0071013 catalytic step 2 spliceosome	60	0.054873
GO:0016564 transcription repressor activity	23	0.054802
GO:0030032 lamellipodium assembly	16	0.054668
GO:0006886 intracellular protein transport	85	0.054646
GO:0042176 regulation of protein catabolic process	10	0.054502
GO:0006363 termination of RNA polymerase I transcription	12	0.054458
GO:0048468 cell development	13	0.054203
GO:0007268 synaptic transmission	47	0.05419
GO:0005980 glycogen catabolic process	12	0.054056
GO:0003746 translation elongation factor activity	15	0.053882
GO:0046966 thyroid hormone receptor binding	22	0.053736
GO:0004003 ATP-dependent DNA helicase activity	21	0.05356
GO:0017148 negative regulation of translation	16	0.053434
GO:0045666 positive regulation of neuron differentiation	17	0.05343
GO:0030048 actin filament-based movement	10	0.053363
GO:0014003 oligodendrocyte development	11	0.053231
GO:0008601 protein phosphatase type 2A regulator activity	11	0.053189
GO:0035255 ionotropic glutamate receptor binding	13	0.053167
GO:0006892 post-Golgi vesicle-mediated transport	30	0.05302
GO:0005978 glycogen biosynthetic process	11	0.052917
GO:0001104 RNA polymerase II transcription cofactor activity	14	0.0529
GO:0004298 threonine-type endopeptidase activity	11	0.052798
GO:0000079 regulation of cyclin-dependent protein kinase activity	21	0.052492
GO:0021766 hippocampus development	12	0.052318
GO:0007264 small GTPase mediated signal transduction	56	0.052213
GO:0005681 spliceosomal complex	26	0.052207
GO:0048365 Rac GTPase binding	13	0.052075
GO:0045177 apical part of cell	26	0.051892
GO:0005525 GTP binding	162	0.051851

GO:0051090 regulation of sequence-specific DNA binding transcription factor activity	14	0.051758
GO:0002756 MyD88-independent toll-like receptor signaling pathway	28	0.051722
GO:0031435 mitogen-activated protein kinase kinase kinase binding	10	0.051672
GO:0033276 transcription factor TFTC complex	14	0.051502
GO:0051087 chaperone binding	19	0.05125
GO:0008584 male gonad development	14	0.051153
GO:0046320 regulation of fatty acid oxidation	10	0.051142
GO:0006096 glycolysis	23	0.051045
GO:0030914 STAGA complex	14	0.050982
GO:0006369 termination of RNA polymerase II transcription	30	0.050923
GO:0016055 Wnt receptor signaling pathway	23	0.050856
GO:0000188 inactivation of MAPK activity	10	0.050829
GO:0034613 cellular protein localization	13	0.050759
GO:0005881 cytoplasmic microtubule	17	0.050695
GO:0030374 ligand-dependent nuclear receptor transcription coactivator activity	27	0.050422
GO:0006626 protein targeting to mitochondrion	13	0.050404
GO:0043034 costamere	15	0.050347
GO:0030175 filopodium	17	0.050326
GO:0005852 eukaryotic translation initiation factor 3 complex	13	0.050104
GO:0051568 histone H3-K4 methylation	12	0.04993
GO:0042277 peptide binding	21	0.049889
GO:0070588 calcium ion transmembrane transport	13	0.049752
GO:0004715 non-membrane spanning protein tyrosine kinase activity	13	0.049637
GO:0031011 Ino80 complex	10	0.049619
GO:0043021 ribonucleoprotein complex binding	11	0.049584
GO:0031145 anaphase-promoting complex-dependent proteasomal ubiquitin-dependent protein catabolic process	56	0.048973
GO:0030326 embryonic limb morphogenesis	13	0.048676
GO:0000502 proteasome complex	21	0.048668
GO:0007026 negative regulation of microtubule depolymerization	10	0.048634
GO:0009408 response to heat	25	0.048544
GO:0005778 peroxisomal membrane	16	0.048436
GO:0032851 positive regulation of Rab GTPase activity	18	0.048327
GO:0051436 negative regulation of ubiquitin-protein ligase activity involved in mitotic cell cycle	48	0.047691
GO:0009880 embryonic pattern specification	13	0.047626
GO:0030501 positive regulation of bone mineralization	11	0.047481
GO:0034138 toll-like receptor 3 signaling pathway	27	0.047337

GO:0030900 forebrain development	20	0.047306
GO:0001937 negative regulation of endothelial cell proliferation	10	0.046481
GO:0005801 cis-Golgi network	16	0.046434
GO:0015992 proton transport	10	0.046408
GO:0031532 actin cytoskeleton reorganization	17	0.046305
GO:0030133 transport vesicle	16	0.045769
GO:0006695 cholesterol biosynthetic process	15	0.045618
GO:0043928 exonucleolytic nuclear-transcribed mRNA catabolic process involved in deadenylation-dependent decay	10	0.045593
GO:0043525 positive regulation of neuron apoptotic process	12	0.045498
GO:0000226 microtubule cytoskeleton organization	26	0.04526
GO:0000186 activation of MAPKK activity	18	0.045252
GO:0002755 MyD88-dependent toll-like receptor signaling pathway	32	0.045194
GO:0034142 toll-like receptor 4 signaling pathway	28	0.045079
GO:0051437 positive regulation of ubiquitin-protein ligase activity involved in mitotic cell cycle	56	0.044921
GO:0034130 toll-like receptor 1 signaling pathway	28	0.044895
GO:0070374 positive regulation of ERK1 and ERK2 cascade	11	0.044767
GO:0008063 Toll signaling pathway	29	0.044667
GO:0030174 regulation of DNA-dependent DNA replication initiation	10	0.044084
GO:0000159 protein phosphatase type 2A complex	11	0.043686
GO:0004402 histone acetyltransferase activity	33	0.043663
GO:0032480 negative regulation of type I interferon production	16	0.043103
GO:0006268 DNA unwinding involved in replication	11	0.043076
GO:0051287 NAD binding	27	0.042779
GO:0001938 positive regulation of endothelial cell proliferation	15	0.042768
GO:0034134 toll-like receptor 2 signaling pathway	29	0.042042
GO:0042809 vitamin D receptor binding	16	0.042038
GO:0042060 wound healing	13	0.04166
GO:0001948 glycoprotein binding	18	0.041362
GO:0016311 dephosphorylation	17	0.041023
GO:0010977 negative regulation of neuron projection development	10	0.040946
GO:0016592 mediator complex	22	0.04075
GO:0004004 ATP-dependent RNA helicase activity	20	0.040743
GO:0031252 cell leading edge	25	0.040381
GO:0003743 translation initiation factor activity	38	0.040372
GO:0007202 activation of phospholipase C activity	10	0.039955
GO:0055038 recycling endosome membrane	11	0.039912
GO:0001756 somitogenesis	20	0.039855
GO:0030838 positive regulation of actin filament polymerization	17	0.039625

GO:0051403 stress-activated MAPK cascade	21	0.039556
GO:0019003 GDP binding	28	0.039378
GO:0005902 microvillus	12	0.039243
GO:0000245 spliceosomal complex assembly	16	0.038887
GO:0000003 reproduction	17	0.038846
GO:0008353 RNA polymerase II carboxy-terminal domain kinase activity	11	0.038762
GO:0051259 protein oligomerization	10	0.038596
GO:0051091 positive regulation of sequence-specific DNA binding transcription factor activity	20	0.038489
GO:0042147 retrograde transport, endosome to Golgi	15	0.038045
GO:0001541 ovarian follicle development	12	0.037812
GO:0006890 retrograde vesicle-mediated transport, Golgi to ER	19	0.037795
GO:0050680 negative regulation of epithelial cell proliferation	14	0.037616
GO:0006977 DNA damage response, signal transduction by p53 class mediator resulting in cell cycle arrest	34	0.037314
GO:0071407 cellular response to organic cyclic compound	12	0.037044
GO:0050690 regulation of defense response to virus by virus	16	0.036436
GO:0003924 GTPase activity	111	0.03607
GO:0006334 nucleosome assembly	22	0.035898
GO:0032880 regulation of protein localization	22	0.035695
GO:0033267 axon part	12	0.035652
GO:0031647 regulation of protein stability	11	0.035491
GO:0030017 sarcomere	19	0.035331
GO:0050768 negative regulation of neurogenesis	10	0.034811
GO:0008139 nuclear localization sequence binding	10	0.033633
GO:0006184 GTP catabolic process	102	0.033382
GO:0017048 Rho GTPase binding	11	0.033207
GO:0010243 response to organic nitrogen	15	0.032194
GO:0019861 flagellum	10	0.032191
GO:0030672 synaptic vesicle membrane	11	0.031354
GO:0006370 7-methylguanosine mRNA capping	20	0.031176
GO:0000786 nucleosome	11	0.030006
GO:0048205 COPI coating of Golgi vesicle	11	0.0299
GO:0007088 regulation of mitosis	10	0.029418
GO:0006469 negative regulation of protein kinase activity	20	0.02677
GO:0004693 cyclin-dependent protein kinase activity	11	0.026274
GO:0051084 'de novo' posttranslational protein folding	20	0.025113
GO:0031529 ruffle organization	10	0.023219
GO:0030530 heterogeneous nuclear ribonucleoprotein complex	14	0.022103
GO:0051258 protein polymerization	17	0.020482
GO:0015631 tubulin binding	10	0.019618
GO:0005680 anaphase-promoting complex	12	0.019151
GO:0005524 ATP binding	655	0.017751

GO:0030057 desmosome	11	0.017407
GO:0005484 SNAP receptor activity	10	0.015567
GO:0016459 myosin complex	10	0.01417
GO:0031581 hemidesmosome assembly	10	0.013895
GO:0032956 regulation of actin cytoskeleton organization	10	0.012927
GO:0004842 ubiquitin-protein ligase activity	118	0.002992
GO:0050796 regulation of insulin secretion	17	0.002734
GO:0043687 post-translational protein modification	79	0.001814
GO:0031625 ubiquitin protein ligase binding	53	0.001267
GO:0006511 ubiquitin-dependent protein catabolic process	56	0.00124
GO:0070936 protein K48-linked ubiquitination	20	0.000848
GO:0070534 protein K63-linked ubiquitination	14	0.000768
