# Supplementary material for: Toward understanding the genetic basis of adaptation to high-elevation life in poikilothermic species: A comparative transcriptomic analysis of two ranid frogs, Rana chensinensis and R. kukunoris
Source: BMC Genomics. 2012 Nov 1;13:588. doi: 10.1186/1471-2164-13-588 (PMC3542248; doi:10.1186/1471-2164-13-588)

Additional file 4. Sampling sites and altitude distribution. Samples of *Rana chensinensis* were collected from the Yaodu Town (105.45°E, 32.79°N) with an altitude of 604 m above sea level (a.s.l.). Samples of *R. kukunoris* were collected from the Zoige County (102.90°E, 33.58°N) with an altitude of 3,358 m a.s.l.

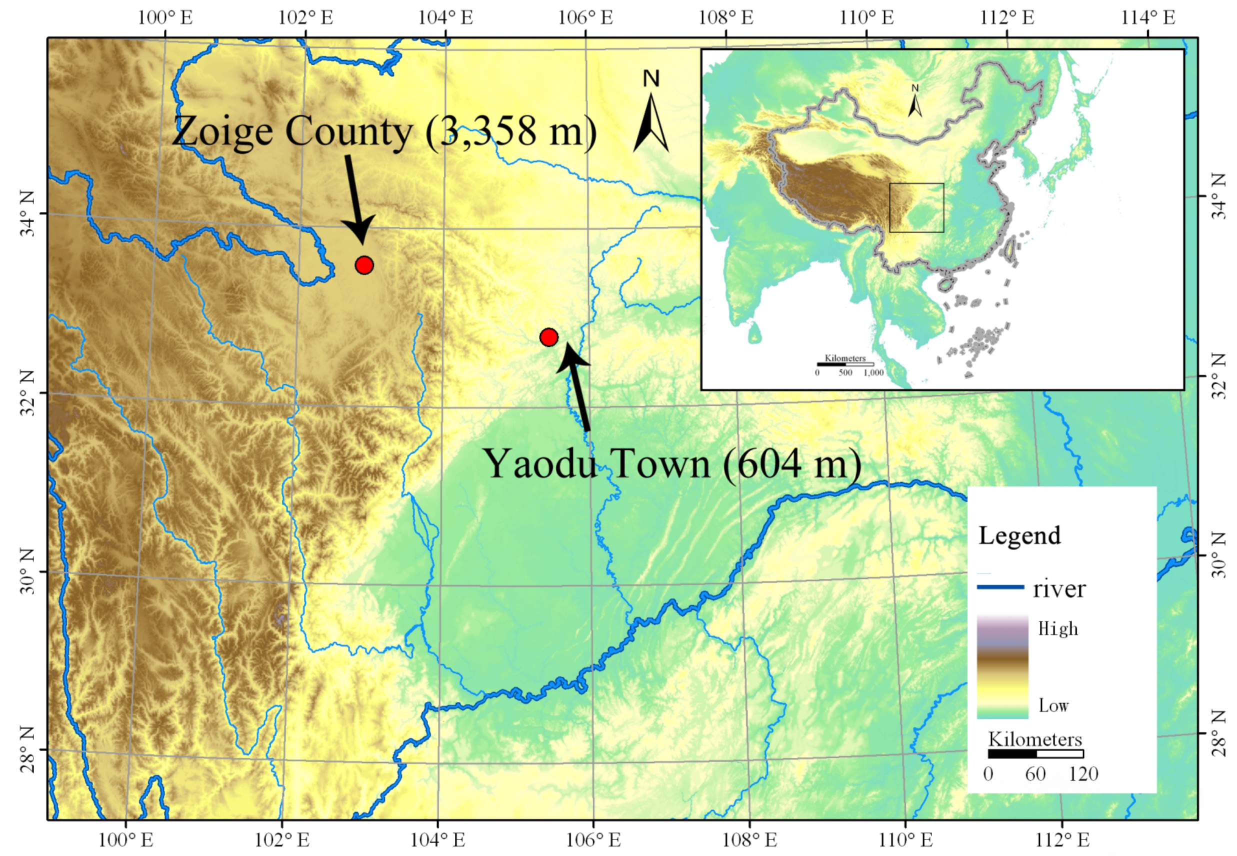

Supplement: Additional file 4 — Sampling sites and altitude distribution. Samples of Rana chensinensis were collected from Yaodu Township (105.45°E, 32.79°N) with an altitude of 604 m above sea level. Samples of R. kukunoris were collected from Zoige County (102.90°E, 33.58°N) with an altitude of 3,358 m above sea level. [file 1471-2164-13-588-S4.pdf]
